# Role for NF-κB in herpes encephalitis pathology in mice genocopying an inborn error of IRF3-IFN immunity

**DOI:** 10.1084/jem.20250064

**Published:** 2025-10-09

**Authors:** Manja Idorn, Xiangning Ding, Stefanie Fruhwürth, Søren Holste, Line S. Reinert, Christian S. Skoven, Katarina Türner-Stenström, Alexander Schmitz, Mikkel H. Vendelbo, Benedicte P. Ulhøi, Dzeneta Vizlin-Hodzic, Mona Wefelmeyer, Ryo Narita, Lona J. Kroese, Ivo J. Huijbers, Michelle Møhlenberg, Anne Kruse Hollensen, Xin Lai, Marie B. Iversen, Brian Hansen, Trine H. Mogensen, Søren R. Paludan

**Affiliations:** 1 Department of Biomedicine, Aarhus University, Aarhus, Denmark; 2 Center for Immunology of Viral infections, Aarhus University, Aarhus, Denmark; 3 Department of Psychiatry and Neurochemistry, Institute of Neuroscience and Physiology, Sahlgrenska Academy, University of Gothenburg, Göteborg, Sweden; 4 Department Clinical Medicine, Center of Functionally Integrative Neuroscience (CFIN), Aarhus University, Aarhus, Denmark; 5 Department of Nuclear Medicine and PET, Aarhus University Hospital, Aarhus, Denmark; 6 Department of Pathology, Aarhus University Hospital, Aarhus, Denmark; 7 Animal Modeling Facility, Netherlands Cancer Institute, Amsterdam, The Netherlands; 8 Department of Infectious Diseases, Aarhus University Hospital, Aarhus, Denmark; 9 Department of Rheumatology and Inflammation Research, Institute of Medicine, Sahlgrenska Academy, University of Gothenburg, Göteborg, Sweden

## Abstract

Herpes simplex encephalitis (HSE) is a devastating disease with high mortality and serious sequelae. Genetic defects in the IFN-I pathway predispose individuals to HSE, but underlying mechanisms remain unclear. Using transgenic mice with the IRF3 R278Q mutation, ortholog to HSE-associated IRF3 R285Q, and iPSC-derived CNS cells from a pediatric patient carrying the variant, we investigated mechanisms in HSE. IRF3 R278Q transgenic mice exhibited aggravated HSV-1 brain disease and elevated CNS viral loads. Accordingly, microglia from the IRF3 R278Q mice showed reduced HSV-1–induced IFN-I expression. Surprisingly, unaltered *Ifnb* levels along with elevated levels of inflammatory cytokines were detected in infected transgenic mouse brains, correlating with higher viral load. This was successfully modeled in patient microglia. Multiomics-based immune profiling revealed an inflammatory monocyte population in the infected IRF3 R278Q mouse brain, which was enriched for NF-κB activation. NF-κB inhibition improved disease outcomes, surpassing the effect of acyclovir. These findings suggest that IFN-I defects lead to elevated levels of HSV-1 replication in the brain, which subsequently enables NF-κB–driven immunopathology, offering insights with therapeutic potential.

## Teaser

Idorn et al. characterized a mouse strain harboring a mutation identified in an HSE patient. Defective IFN-driven antiviral responses led to hyperactivation of inflammatory responses, which contributed to disease development. The study identifies immunopathology as an important contributor to HSE pathogenesis.

## Graphical Abstract

**Figure:**
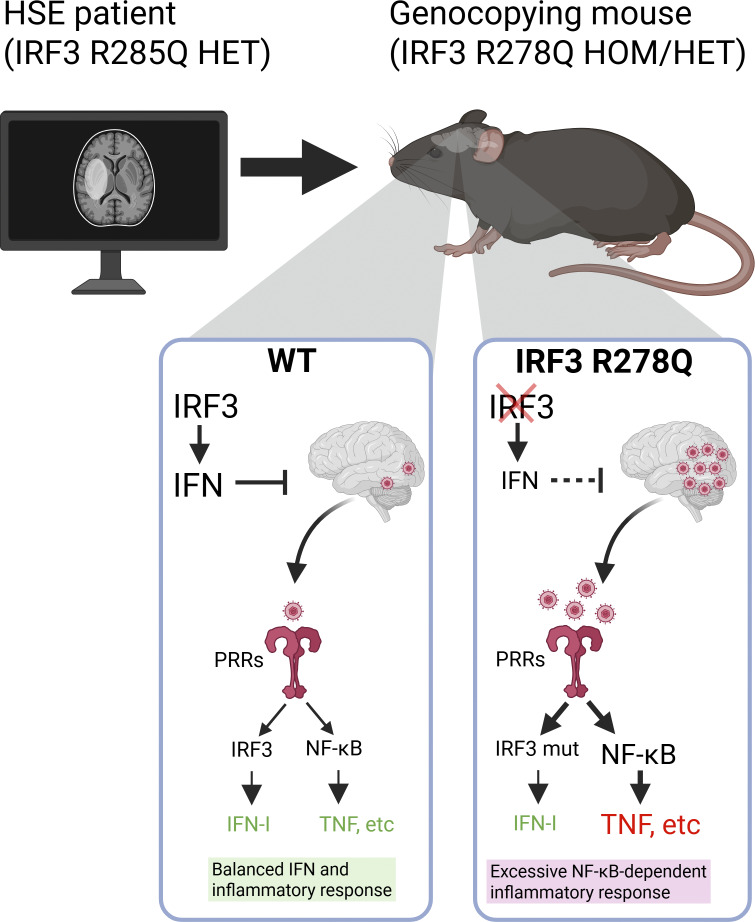

## Introduction

Herpes simplex encephalitis (HSE), which is caused by HSV type 1 (HSV-1) infection in the brain, is a devastating disease with high mortality if not treated early with antiviral therapy, and even with treatment, mortality and morbidity is considerable (Stahl and Mailles, 2019). Pathological examination of postmortem brains from HSE patients have revealed profound brain pathology with focal necrosis and infiltrating leukocytes (Mendez et al., 2018; Miyake et al., 1977). HSV-1 is a neurotropic virus initiating infection in epithelial cells on mucosal surfaces, allowing downstream infection of innervating sensory neurons (Roizman et al., 2007). In rare cases, HSV-1 spreads further to the CNS through the neuronal route and can give rise to HSE (Whitley, 2015). Most cases of HSE in children occur during primary infection, whereas the majority of cases of HSE in adults occur during reactivation (Stahl and Mailles, 2019).

The innate immune response contributes to both early host defense against infection and pathological inflammation (Mogensen, 2009). This response is evoked by pattern recognition receptors (PRRs), which are activated upon sensing of microbial molecules. The main PRR-induced antiviral mechanism is mediated by type I IFNs, which can exert direct blockage of virus replication through induction of IFN-stimulated genes (ISGs) with virus-restricting activity (Dalskov et al., 2023). The proinflammatory activities are induced via cytokines, such as TNFα, IL 1β, and IL-6, and involve a series of effector mechanisms, including recruitment of leukocytes, release of degenerative enzymes, and induction of cell death (Li and Wu, 2021). Cell culture studies have identified and described in molecular details that HSV-1 infection is sensed by and can activate signaling from TLR2, TLR3, TLR9, RIG-I, MDA5, cGAS, AIM2, and NLRP3 (Johnson et al., 2013; Krug et al., 2004; Kurt-Jones et al., 2004; Maruzuru et al., 2018; Melchjorsen et al., 2010; Reinert et al., 2016; Zhang et al., 2007; Zhao et al., 2016) in different cell types. PRR activation and downstream signaling leads to activation of the transcription factor IFN regulator factor 3 (IRF3) and induction of ISG expression, as well as activation of the transcription factor NF-κB and induction of a strong proinflammatory gene expression program (Li and Wu, 2021; Mogensen, 2009). All known signaling PRRs can activate NF-κB, and a subset can also activate IRF3-IFN signaling, including TLR3, RIG-I, MDA5, and cGAS.

Studies in mice with full gene deletion have shown that type I IFN is essential for control of HSV-1 in the brain under experimental conditions. cGAS-STING signaling in microglia plays an important role for early induction of IFN expression (Katzilieris-Petras et al., 2022; Reinert et al., 2016), while IFN receptor signaling in neurons and astrocytes is essential for exerting the antiviral activity (Hayes et al., 2022; Rosato and Leib, 2015). By contrast, mice with defects in TLR2 or the NLRP3 adaptor protein ASC are partly protected from developing HSE-like disease (Hayes et al., 2021; Kurt-Jones et al., 2004). Importantly, these mice show reduced expression levels of inflammatory cytokines and impaired influx of leukocytes to the CNS. Thus, a fine-balanced innate immune response is important to control HSV-1 infection during acute infection in the brain, but the mechanisms that govern this balance, and their disturbance in HSE, remain poorly understood.

Within the last 10–15 years, studies of rare gene variants in HSE patients have led to profound advances in the understanding of the genetic requirements for protection from HSE in humans (Zhang and Casanova, 2024). Most identified variants are loss-of-function for production and function of type I IFNs. These include TLR3, UNC93B1, TRIF, TRAF3, TBK1, NEMO, IRF3, IFNAR1, STAT1, and STAT2 (Andersen et al., 2015; Audry et al., 2011; Bastard et al., 2021; Bucciol et al., 2023; Casrouge et al., 2006; Dupuis et al., 2003; Herman et al., 2012; Pérez de Diego et al., 2010; Sancho-Shimizu et al., 2011; Zhang et al., 2007). Of these, Andersen et al. (2015) reported a pediatric HSE patient carrying a heterozygous loss-of-function IRF3 R285Q mutation. PBMC and fibroblasts from this patient exhibited reduced induction of *IFNB* expression upon HSV-1 infection. This cellular phenotype was rescued upon reintroduction of the IRF3 WT allele into patient fibroblasts. These data strongly suggested a causal link between the genotype and the phenotype. However, although our knowledge on what predisposes to HSE has expanded significantly, we still have very limited knowledge on the mechanisms that drive the development and progression of the disease in infected individuals with inborn errors of IFN immunity (IEI).

In this work, we generated a transgenic mouse carrying the ortholog of the human IRF3 R285Q mutation to model the previously identified IEI in humans *in vivo* to uncover central mechanisms in the pathogenesis of HSE. Unlike full knockout mice, such transgenic mice resemble the complex situation of the patients much better. This includes expression of proteins of interest with potentially altered functions, such as partial loss-of-function, dominant-negative activity, etc. We show that these mice exhibit inability to control HSV-1 following corneal infection, thus leading to increased viral inoculum into the CNS. This induced an increased expression of inflammatory cytokines by microglia driven by NF-κB. This in turn enabled downstream influx and activation of a specific monocyte subpopulation with strong activation of IFNγ- and NF-κB–induced proinflammatory mediators. Finally, blocking the NF-κB response led to reduced inflammatory response and ameliorated disease symptoms. Our work suggests that early disbalanced antiviral versus inflammatory gene expression in the virus-infected brain fuels NF-κB–driven immunopathology in HSE.

## Results

### The IRF3 R285Q mutation impairs type I IFN responses in microglia and confers susceptibility to disease in a HSE mouse model

**Figure 1.**
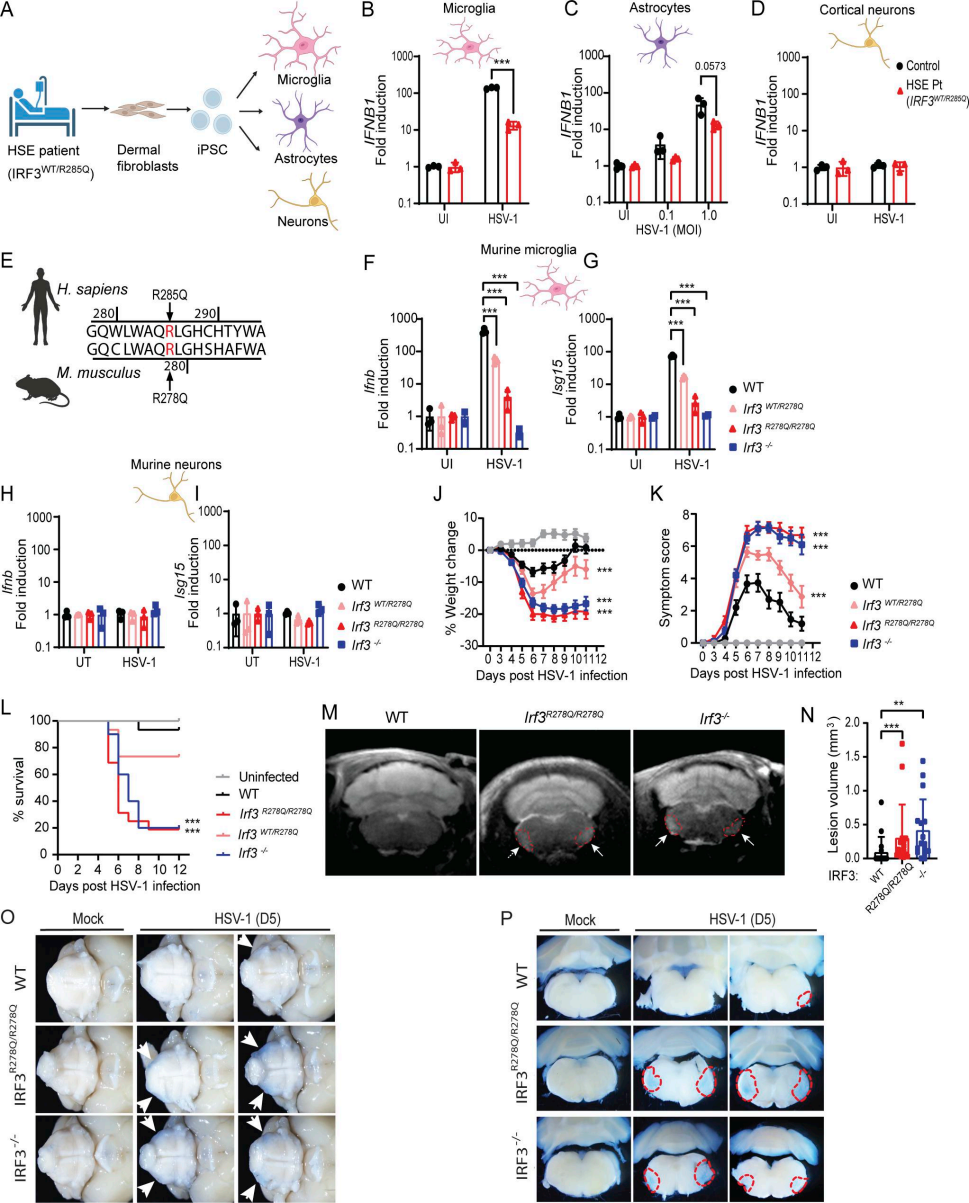
**The hIRF3 R285Q/mIRF3 R278Q mutation impairs type I IFN responses in microglia and confers susceptibility to HSE-like disease. (A)** Human iPSC-derived microglia, neurons, and astrocytes were generated from patient fibroblasts or control iPSC. Created with BioRender. **(B–D)***IFNB1* expression in (B) microglia, (C) astrocytes, and (D) cortical neurons 24 h after infection with HSV-1 at MOI 1.0. HSE pt., HSE patient. **(E)** Alignment of human and murine IRF3 around the region harboring R285 in WT human IRF3. **(F–I)***Ifnb1* and *Isg15* expression after HSV-1 infection in murine microglia and neurons from WT and transgenic mice carrying the IRF3 R278Q mutation. Microglia (F and G) and neurons (H and I) 24 h after infection with HSV-1 at MOI 1.0. All *in vitro* experiments were performed in triplicates and independently repeated at least three times. Expression data were normalized to β-actin and shown as fold change compared with the UI control. **(J–O)** Mice were infected in the cornea with HSV-1 McKrea (2 × 10^6^ PFU/eye), and HSE-like disease development was followed over time until reaching humane endpoint or recovering 100% of starting weight. **(J)** % weight change. **(K)** Symptom score. **(L)** Survival curve (UI, *n* = 7; WT, *n* = 15; *Irf3*^*WT/R278Q*^, *n* = 15; *Irf3*^*R278Q/R278Q*^, *n* = 16; *Irf3*^*−/−*^*n* = 10). Dead animals were censored in the graphs and thus represented in the graphs with weight and symptom score at time of death. **(M)** Representative MR images performed on day 5 after infection. Red dotted line and white arrows indicate lesions. **(N)** Lesion volumes quantified blinded. **(O and P)** BBB disruption/integrity was assessed visibly by Evans blue perfusion of mice 5 days after HSV-1 infection. Representative microscope images of Evans blue dye leakage in brain stems from UI and HSV-1–infected WT and IRF3^R278Q/R278Q^ mice were obtained from (O) uncut ventral position (2× objective) and (P) coronal slides cut in 5 mm thickness (3.2× objective). Red circles indicate area of Evans blue passive diffusion into lesion sites. *n* = 3–7 mice per group. *In vivo* survival experiments were independently repeated three times, and MR-imaging experiment was repeated two times. Statistical analyses of cell culture experiments (B–D and F–I) were analyzed by two-tailed two-way ANOVA for difference of means, followed by two-tailed unpaired *t* test of means, error bars; SD. Disease development (weight change and symptom score) were compared between the groups using a mixed-effects analysis with Geisser-Greenhouse correction for multiple interacting variables (time and genotype). Survival was analyzed using log-rank Mantel–Cox test (L). Error bars; SEM. Lesion volumes (N) were analyzed by two-tailed one-way ANOVA followed by unpaired *t* test, error bars; SD. P values <0.05 were considered statistically significant, **P < 0.01, and ***P < 0.001.

**Figure S1.**
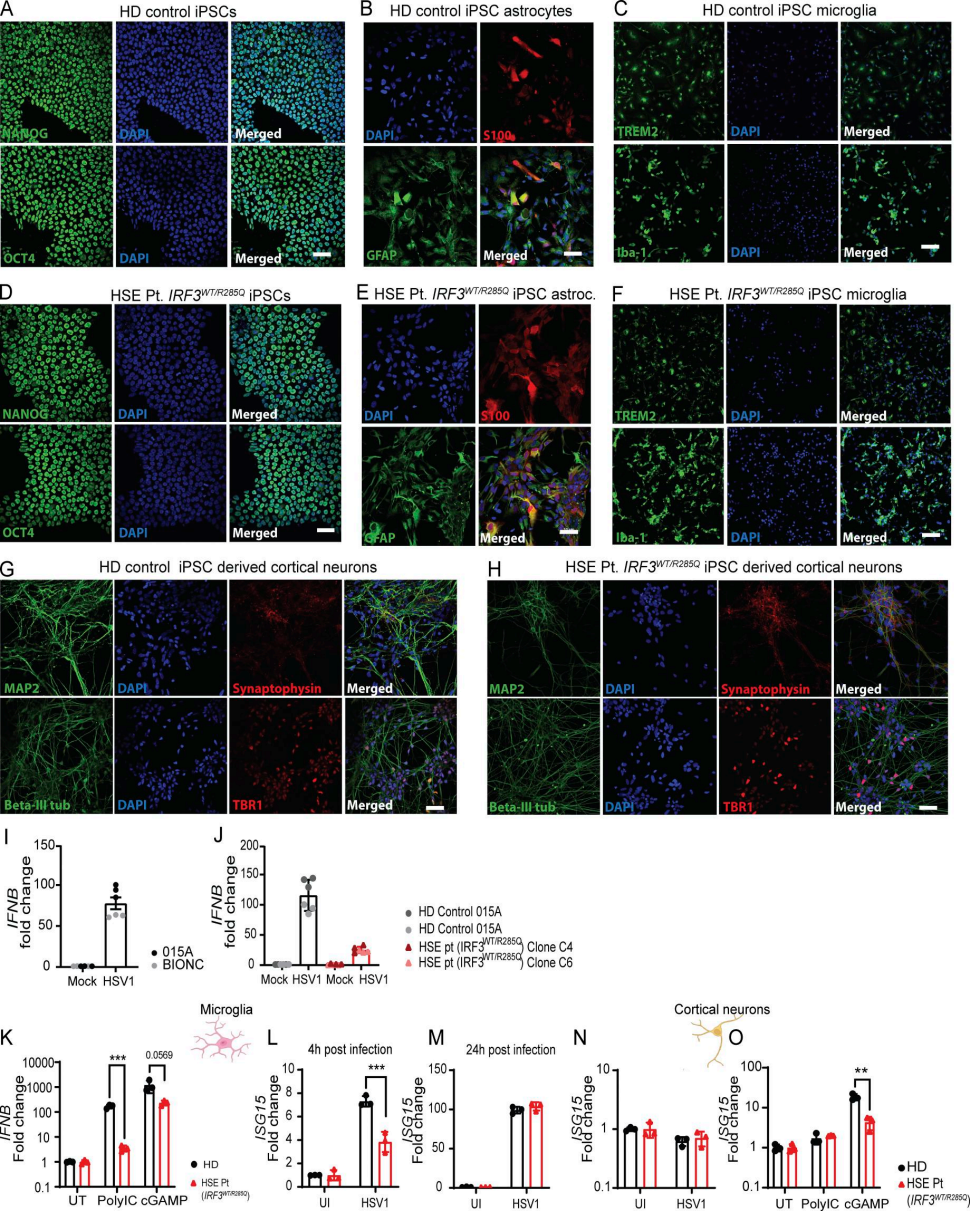
**Generation of human iPSC-derived microglia, cortical neurons, and astrocytes. (A–H)** Confocal microscopy images of iPSCs and derived astrocytes, microglia, and cortical neurons from a healthy control donor and a pediatric HSE patient heterozygous for the IRF3 R285Q amino acid substitution stained with cell type–specific markers. (A and D) iPSCs: NANOG and OCT4 (both green); (B and E) astrocytes: S100 (red) and GFAP (green); (C and F) microglia: TREM2 and Iba1 (both green); (G and H) cortical neurons: MAP2, β-III tubulin (both green), synaptophysin, and TBR1 (both red); nuclei were identified by DAPI staining. Scale bar, 50 μm. **(I and J)** Comparison of *IFNB* response to HSV-1 infection in microglia from two different iPSC lines and two different patient-derived iPSC clones measured by RT-qPCR. Commercially available iPSC lines 015A and BIONC (I) and patient-derived iPSC clones C4 and C6 (J). **(K)***IFNB* expression in microglia after stimulation with polyIC (25 μg/ml) and cGAMP (100 μg/ml). **(L and M)***ISG15* expression in microglia 4 and 24 h after infection with HSV-1 at MOI 1.0. **(N and O)***ISG15* expression in cortical neurons infected 24 h with HSV-1 at MOI 1.0 or stimulated for 4 h with polyIC (25 μg/ml) and cGAMP (100 μg/ml). Expression data were normalized to β-actin and shown as fold change compared with the UI control. Statistical analyses of gene expression in CNS cell cultures (K–O) were analyzed by two-tailed two-way ANOVA for difference of means, followed by an unpaired *t* test of means, error bars; SD. P values <0.05 were considered statistically significant, **P < 0.01, and ***P < 0.001.

To investigate how the IRF3 R285Q mutation contributes to the pathogenesis of HSE, we first examined the innate immune response of brain cells derived from an adolescent HSE patient carrying this mutation, as previously reported (Andersen et al., 2015). Skin fibroblasts from the patient were reprogrammed to generate induced pluripotent stem cells (iPSCs) (Fig. 1 A and Fig. S1, A and D). iPSCs from the HSE patient and healthy donors were differentiated into microglia, astrocytes, and cortical neurons (Fig. S1, B, C, and F–H), and the induction of the type I IFN gene *IFNB* and the ISG *ISG15* were assessed (Fig. S1, I and J). Patient-derived microglia showed reduced, but not abrogated, induction of *IFNB* in response to PRR agonists poly(I:C) and cGAMP (Fig. S1 K). Upon HSV-1 infection, HSE patient microglia showed an impaired *IFNB* response (Fig. 1 B and Fig. S1 J), which was accompanied by reduced early but not late expression of *ISG15* (Fig. S1, L and M). We observed a similar tendency toward defective *IFNB* response to HSV-1 infection in HSE patient astrocytes compared with control astrocytes (Fig. 1 C). Regarding neurons, human iPSC-derived cortical neurons did not induce *IFNB* or *ISG15* in response to HSV-1 infection (Fig. 1 D and Fig. S1 N), although *ISG15* was modestly induced by the STING agonist cGAMP (Fig. S1 O). Consistent with the lack of IFN-ISG response to HSV-1 infection, patient neurons did not differ from the control neurons on this parameter.

**Figure S2.**
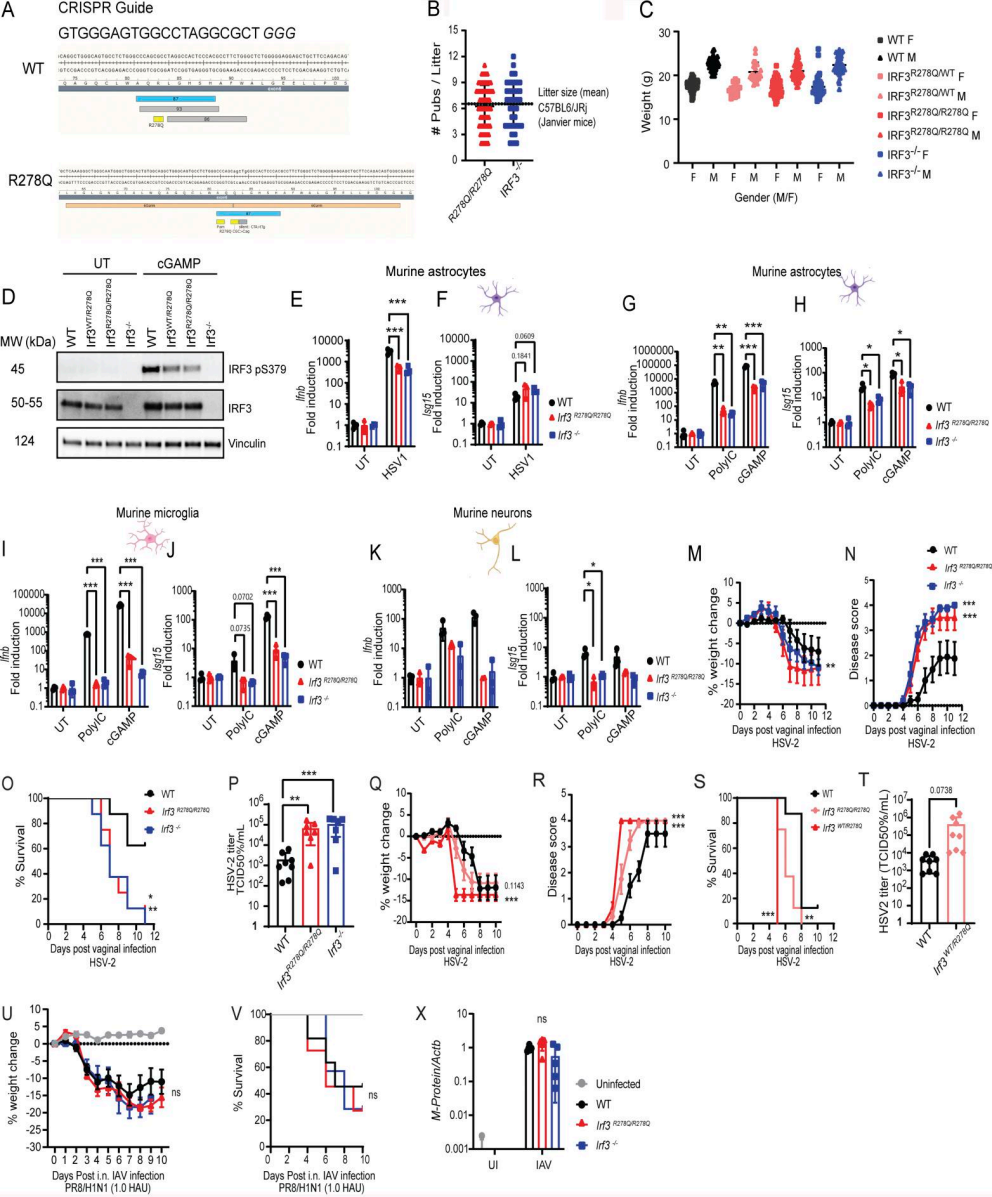
**Generation and characterization of transgenic mice carrying the IRF3 R278Q allele and susceptibility to infections with HSV-2 and IAV. (A)** Mice carrying the patient-specific mIRF3 R278Q amino acid substitution were made using CRISPR microinjection in C57Bl6/J zygotes. The CRISPR guide used was 5′-GTG​GGA​GTG​GCC​TAG​GCG​CTG​GG-3′. **(B)** Litter size of *Irf3*^*R278Q/R278Q*^ and *Irf3*^*−/−*^ pubs bred at the Aarhus University animal core facility in 2024 compared with average litter size of C57Bl6/JRj mice bred by Janvier. **(C)** Weight of C57Bl6/JRj, *Irf3*^*WT/R278Q*^, *Irf3*^*R278Q/R278Q*^, and *Irf3*^*−/−*^ mice at experiment start (square, female; triangle, male). **(D)** WB analysis of IRF3 protein and phosphorylation of IRF3 Ser379 in lysates of BMDMs from WT, *Irf3*^*WT/R278Q*^, *Irf3*^*R278Q/R278Q*^, and *Irf3*^*−/−*^ mice stimulated with 100 μg/ml cGAMP for 2 h. **(E and F)***Ifnb* and *Isg15* gene expression response of murine astrocyte cultures to HSV-1 infection at MOI 1.0 for 24 h. **(G–L)***Ifnb* and *Isg15* gene expression response to stimulation with PRR agonists poly-IC (25 μg/ml) or cGAMP (100 μg/ml) for 4 h. Murine astrocyte (G and H), murine microglia (I and J), and murine neurons (K and L). CNS cell culture gene expression was measured by RT-qPCR, and data were normalized to β-actin (*Actb*) and are represented as fold change normalized to expression in UI control. All *in vitro* experiments were performed in triplicates and independently repeated at least three times. Statistical analyses of gene expression in CNS cell cultures (E–L) were analyzed by two-tailed two-way ANOVA for difference of means, followed by an unpaired *t* test of means, error bars; SD. **(M–X)** WT, *Irf3*^*WT/R278Q*^, *Irf3*^*R278Q/R278Q*^, and *Irf3*^*−/−*^ mice were infected with (M–T) HSV-2 by the vaginal route or (U–X) IAV via the nasal route and were followed for disease development over time until reaching humane endpoint or recovering 100% of starting weight. **(M, Q, and U)** % weight change. **(N and R)** Symptom score. **(O, S, and V)** Survival curve. Dead animals were censored in the graphs and thus represented in the graphs with weight and symptom score at time of death. HSV-2 longitudinal (M–O) (WT, *n* = 8; *Irf3*^*R278Q/R278Q*^, *n* = 8; *Irf3*^*−/−*^*n* = 8), (Q–S) (WT, *n* = 8; *Irf3*^*WT/R278Q*^, *n* = 8; *Irf3*^*R278Q/R278Q*^, *n* = 8), and IAV longitudinal (U and V) (WT, *n* = 11; *Irf3*^*R278Q/R278Q*^, *n* = 11; *Irf3*^*−/−*^, *n* = 7; UI, *n* = 6). Viral load was assessed by (P and T) HSV-2 TCID50% assay of vaginal washes on day 2 after infection; (P) WT, *n* = 8; *Irf3*^*R278Q/R278Q*^, *n* = 7; *Irf3*^*−/−*^*n* = 7; and (T) WT, *n* = 8; *Irf3*^*WT/R278Q*^, *n* = 8; or (X) *IAV M-Protein* gene transcripts in lung homogenates on day 4 postnasal inhalation infection measured by RT-PCR (WT, *n* = 5; *Irf3*^*R278Q/R278Q*^, *n* = 5; *Irf3*^*−/−*^*n* = 5). Disease development (M, N, Q, R, and U) was compared between the groups using a mixed-effects analysis with Geisser-Greenhouse correction for multiple interacting variables (time and genotype). Error bars; SEM. Survival (O, S, and V) was analyzed using log-rank Mantel–Cox test. Viral load (P, T, and X) was analyzed by two-tailed two-way ANOVA for difference of means, followed by an unpaired *t* test of means, error bars; SD. P values <0.05 were considered statistically significant, *P < 0.05, **P < 0.01, and ***P < 0.001. Source data are available for this figure: SourceData FS2.

To study the pathological mechanisms of the IRF3 R285Q mutation, we generated a transgenic mouse strain carrying the orthologous R278Q mutation in IRF3 (Fig. 1 E and Fig. S2 A). Unlike full knockout mice, transgenic mice express the mutant protein, allowing us to study the impact of residual or dominant-negative activities on disease development, better mirroring the complex pathophysiological situation in HSE patients. Transgenic mice had normal litter sizes and weight development compared with WT mice (Fig. S2, B and C). Expression levels of IRF3 were not altered (Fig. S2 D), but we observed impaired IRF3 phosphorylation on Ser379 (equivalent to Ser386 in human IRF3) in response to agonist stimulation (Fig. S2 D), in agreement with previous reports (Andersen et al., 2015; Dalskov et al., 2020). *In vitro* examination of murine microglia heterozygous for the IRF3 R278Q mutation mirrored the impaired *Ifnb* response to HSV-1 in microglia from the HSE patient, and this was even more pronounced in homozygous *Irf3*^*R278Q/R278Q*^ murine microglia (Fig. 1, F and G). Interestingly, however, microglia carrying the transgene showed residual *Ifnb* induction, which was also observed in cells from the patient (Andersen et al., 2015). In comparison, IRF3-deficient (*Irf3*^*−/−*^) microglia completely failed to induce *Ifnb* upon HSV-1 infection *in vitro*. Astrocytes from *Irf3*^*R278Q/R278Q*^ mice also showed reduced expression of *Ifnb* (Fig. S2, E and F). Finally, like human cortical neurons, murine neurons did not elevate *Ifnb* expression upon HSV-1 infection *in vitro* (Fig. 1, H and I). To complete the broad characterization of type I IFN responses in brain cells from transgenic mice, we stimulated with PRR agonists poly(I:C) and cGAMP and found that microglia, astrocytes, and neurons all showed an impaired type I IFN response (Fig. S2, G–L). Thus, iPSC-derived microglia and astrocytes from the HSE patient with the heterozygous IRF3 R285Q mutation show impaired HSV-1–induced IFN expression, and this is largely phenocopied in the same cell types from transgenic mice carrying the IRF3 R278Q mutation.

We next infected mice with HSV-1 via an ocular route to induce HSE-like disease. Mice homozygous for *Irf3*^*R278Q/R278Q*^ developed severe HSE-like disease within 5–6 days compared with WT mice, measured by significant weight loss and increased disease symptom score (Fig. 1, J and K). Disease severity in *Irf3*^*R278Q/R278Q*^ mice were comparable with that observed in *Irf*3^−/−^ mice. Heterozygous *Irf3*^*WT/R278Q*^ mice likewise developed more severe disease compared with WT mice, although not as pronounced as *Irf3*^*R278Q/R278Q*^. When monitoring for survival, we observed that *Irf3*^*R278Q/R278Q*^ mice showed significantly increased risk of death after HSV-1 infection, while the same tendency for the heterozygous *Irf3*^*WT/R278Q*^ mice did not reach statistical significance (Fig. 1 L). The HSE-like disease in the HSV-1–infected mice could be visualized by MRI scans showing lesions in the right and left side of the brain stems (Fig. 1 M). Elevated lesion volumes were observed in infected *Irf3*^*R278Q/R278Q*^ and *Irf3*^*−/−*^ mice compared with WT (Fig. 1 N). In addition, visualized blood–brain barrier (BBB) breakdown using Evans blue injections revealed a greater passive diffusion of the blue dye into the affected areas in the brain stems of HSV-1–infected *Irf3*^*R278Q/R278Q*^ and *Irf3*^*−/−*^ mice compared with WT (Fig. 1, O and P). To test for specificity for HSV-1 infection, we also infected the mice with HSV-2 through the genital route and with influenza A virus (IAV) through the intranasal route. While both homozygous *Irf3*^*R278Q/R278Q*^ and heterozygous *Irf3*^*WT/R278Q*^ mice showed elevated susceptibility to HSV-2 compared with WT mice, no effect of the mutation was observed after IAV infection (Fig. S2, M–X). Of note, the HSE patient with the IRF3 R285Q mutation did not have a clinical history of severe IAV infections (Andersen et al., 2015). Collectively, the IRF3 R278Q mutation renders mice susceptible to HSE-like disease and show shared cellular and phenotypic features with fibroblasts and PBMCs from the HSE patient carrying the IRF3 R285Q mutation (Andersen et al., 2015).

### *Irf3* ^*R278Q/R278Q*^ mice exhibit elevated viral load in the CNS

**Figure 2.**
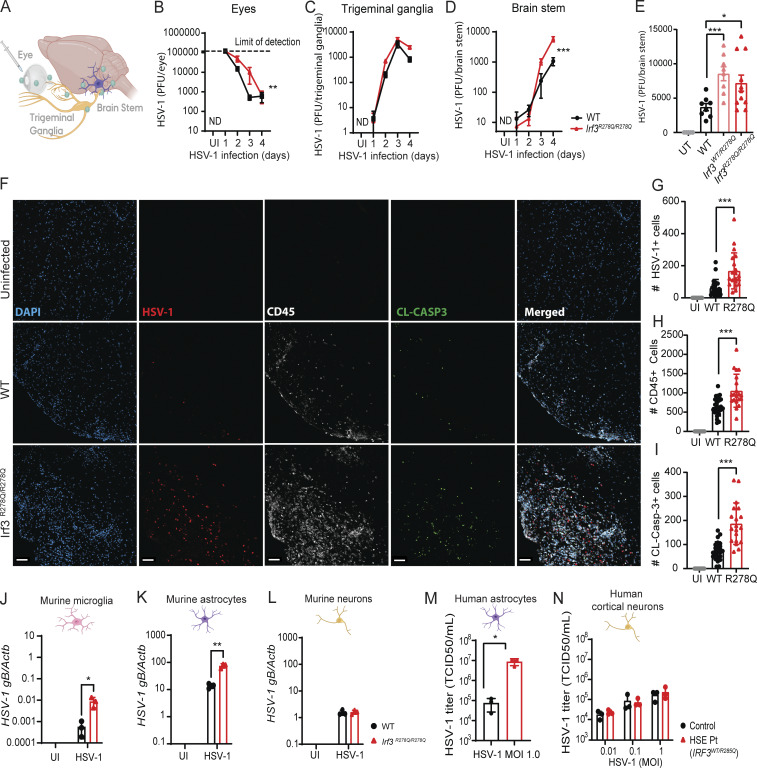
***Irf3*** ^***R278Q/R278Q***^ **mice exhibit elevated viral load in the CNS. (A)** Schematic of the ocular infection route and establishment of HSE-like disease in mice. Image was created in BioRender. Virus replicates in the trigeminal ganglion with spreading to the brain stem. **(B–D)** Eyes (B), TG (C), and brain stems (D) were harvested on the indicated time points from WT (*n* = 5–7) and *Irf3*^*R278Q/R278Q*^ (*n* = 5–12) mice infected with HSV-1 McKrea (2 × 10^6^ PFU/eye). Viral titer determined by plaque assay on tissue homogenates. **(E)** Viral titer in brain stem homogenates isolated on day 5 from WT (*n* = 8), *Irf3*^*WT/R278Q*^ (*n* = 8), and *Irf3*^*R278Q/R278Q*^ (*n* = 11) mice infected with HSV-1 McKrea (2 × 10^6^ PFU/eye). **(F)** Representative confocal microscopy images of HSV-1 foci in the brain stems of WT and *Irf3*^*R278Q/R278Q*^ on day 5 after infection (blue: DAPI, red: HSV-1 ICP5, white: CD45, and green: cleaved-caspase 3). Magnification, 10×; scale bar, 50 μm. **(G–I)** Quantification of images from Fig. 2 E of number of (G) HSV-1 ICP5^+^, (H) CD45^+^, and (I) cleaved caspase-3^+^ cells per image (UI, *n* = 16; WT, *n* = 24; *Irf3*^*R278Q/R278Q*^, *n* = 20 images, respectively) Brain stems imaged: UI, *n* = 4; WT, *n* = 6; *Irf3*^*R278Q/R278Q*^, *n* = 5. Each brain stem is represented by images of two different anatomic locations (−5.80 mm and −6.34 mm, relative to bregma, respectively), two images per brain stem: left and right side lesions, respectively. Scale bar, 50 μm. **(J–L)** Levels of *HSV-1 gB* transcripts 24 h after infection (MOI 1.0) *in vitro* in murine (J) microglia, (K) astrocytes, and (L) neurons were assessed by RT-qPCR of HSV-1 *gB* normalized to β-actin (*Actb*). **(M and N)** HSV-1 titers in supernatants harvested from human iPSC-derived (M) astrocytes and (N) cortical neurons 24 h after infection with MOI 0.01–1, assessed by TCID50% assay. Viral replication kinetics (B–D) were compared between the groups using a mixed-effects analysis with Geisser-Greenhouse correction for multiple interacting variables (time and genotype). Error bars; SEM. Statistical analyses of virus titer, image quantification, and CNS cell experiments (E, G–I, and J–N) were performed by two-tailed one-way ANOVA, followed by unpaired *t* test of means, error bars; SD. P values <0.05 were considered statistically significant, *P < 0.05, **P < 0.01, and ***P < 0.001; N.D., not detected; CL-Casp3, cleaved caspase-3.

**Figure S3.**
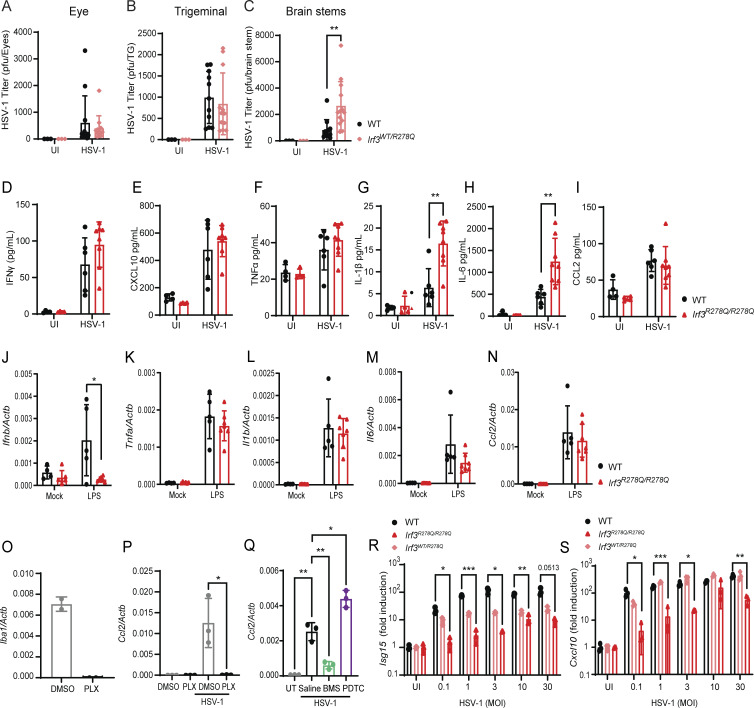
**Comparative analysis of systemic and CNS responses to HSV-1 infection *in vivo* and *in vitro*. (A–C)** Analysis of viral replication on day 4 after HSV-1 ocular infection (2 × 10^6^ PFU/eye) of WT (*n* = 12) and *Irf3*^*WT/R278Q*^ heterozygous (*n* = 12) mice. UI controls were included (WT UI, *n* = 3; *Irf3*^*WT/R278Q*^ UI, *n* = 3). Viral titer in (A) eyes, (B) TG, and (C) brain stem homogenates quantified by plaque assay. **(D–I)** Systemic cytokine levels measured in serum from WT and *Irf3*^*R278Q/R278Q*^ mice on day 5 after HSV-1 infection. Data points represent cytokine level (pg/ml) in serum from individual mice measured by mesoscale. **(J–N)** CNS response in WT and *Irf3*^*R278Q/R278Q*^ mice to systemic LPS stimulation *in vivo*. Gene expression of *Ifnb*, *Tnfa*, *Il1b*, *Il6*, and *Ccl2* in whole-brain homogenates from mice treated with 5 mg/kg LPS or saline by i.p. injection. Gene expression was measured by RT-qPCR, and data were normalized to β-actin (*Actb*). **(O)** Validation of microglia depletion by qPCR of *Iba1* expression in MBCs treated with 0.5 μM PLX5622 (PLX) or untreated (UT). **(P and Q)** Expression of *Ccl2* in WT mixed brain cultures treated with (P) 0.5 μM PLX5622 (PLX) or mock treated for microglia depletion, or (Q) NF-κB activation inhibitors BMS-345541 2 µM, PDTC 25 µM, or saline, and infected with HSV-1 at MOI 1.0 for 24 h. **(R and S)***Isg15* and *Cxcl10* infection-dose response analysis in murine microglia analyzed by RT-qPCR 24 h after infection with HSV-1 at increasing MOI. For all panels where statistical analyses were performed, the analysis was two-tailed two-way ANOVA for difference of means, followed by two-tailed unpaired *t* test of means, error bars; SD. P values <0.05 were considered statistically significant. *P < 0.05, **P < 0.01, and ***P < 0.001.

To further elucidate the effects of the R-to-Q amino acid substitution in IRF3 on mechanisms of HSE pathogenesis, we focused on homozygous *Irf3*^*R278Q/R278Q*^ mice and sought to confirm key findings in the *Irf3*^*WT/R278Q*^ mice. First, the progression of HSV-1 infection from the eye, through the trigeminal ganglia (TG), to the brain stem after ocular infection was monitored (Fig. 2 A). *Irf3*^*R278Q/R278Q*^ mice showed a delayed control of viral replication in the eyes compared with WT mice (Fig. 2 B), which was reflected in a tendency toward overall more virus in the TG (Fig. 2 C). Importantly, in the brain stem, we observed close to 10 times more virus on days 3 and 4 after infection in the *Irf3*^*R278Q/R278Q*^ mice compared with WT (Fig. 2 D). Importantly, the heterozygous *Irf3*^*WT/R278Q*^ mice also exhibited elevated viral load in the brain stem after HSV-1 infection, but not in the eye or TG (Fig. 2 E and Fig. S3, A–C). Using immunofluorescent staining and confocal microscopy, we observed larger infection foci and more virus-positive cells in the brain stem of *Irf3*^*R278Q/R278Q*^ mice compared with WT (Fig. 2, F and G). In addition, CD45^+^ cells were recruited to the HSV-1 lesions (Fig. 2 F), with CD45^+^ cells reflecting infiltrating immune cells, since microglia did not stain positive for CD45^+^ in our hands. The infection foci were also positive for the apoptosis marker cleaved caspase 3 (Fig. 2 F). For both parameters, *Irf3*^*R278Q/R278Q*^ mice showed higher levels than WT mice (Fig. 2, H and I).

To assess HSV-1 replication in CNS cells, HSV-1 *gB* transcripts from the *in vitro*–cultured microglia, astrocytes, and neurons from WT and *Irf3*^*R278Q/R278Q*^ mice (Fig. 1, F–I) were quantified. We found higher levels of HSV-1 *gB* mRNA in *Irf3*^*R278Q/R278Q*^ microglia and astrocytes compared with WT, although microglia did not support HSV-1 replication very well (Fig. 2, J and K). This increase in viral transcription was not observed in neuron cultures (Fig. 2 L). The pattern with increased HSV-1 replication in astrocytes but not neurons was consistent with iPSC-derived cells from the HSE patient (Fig. 2, M and N), in which we did not observe any notable *Ifnb* response to HSV-1 infection (Fig. 1, B–D).

Taken together, these findings suggest that the IRF3 R278Q loss-of-function mutation leads to impaired control of HSV-1 both in the periphery and the CNS, which enables development of HSE.

### Elevated levels of proinflammatory cytokines in the brain of *Irf3*^*R278Q/R278Q*^ mice upon HSV-1 infection

**Figure 3.**
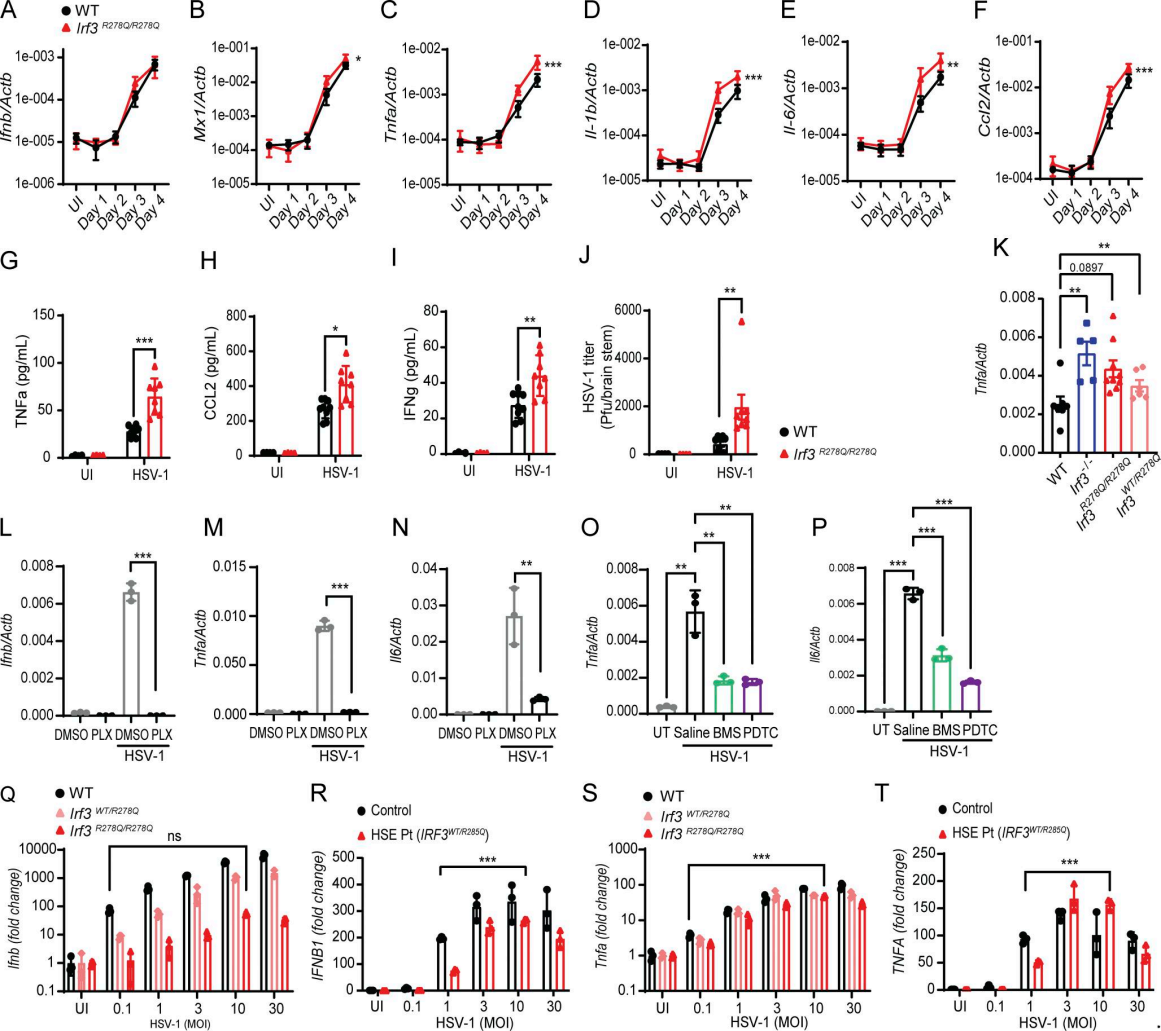
**Elevated proinflammatory cytokine levels in the brain of *Irf3*** ^***R278Q/R278Q***^ **mice upon HSV-1 infection. (A–F)** Expression of *Ifnb*, *Mx1*, *Tnfa*, *Il1b*, *Il6*, and *Ccl2* in brain stems from WT or *Irf3*^*R278Q/R278Q*^ mice on days 1–4 after infection, measured by RT-qPCR. UI, uninfected control. WT, *n* = 5–7; *Irf3*^*R278Q/R278Q*^, *n* = 5–12. **(G–J)** Mesoscale analysis of cytokine levels (TNFα, CCL2, and IFNγ) (G–I) and HSV-1 titer in brain stem homogenates on day 5 after infection quantified by plaque assay (J) (WT UI, *n* = 4, WT, *n* = 9; *Irf3*^*R278Q/R278Q*^ UI, *n* = 4; *Irf3*^*R278Q/R278Q*^, *n* = 8). **(K)** Expression of *Tnfa* in brain stems from WT (*n* = 7), *Irf3*^*−/−*^(*n* = 5), *Irf3*^*R278Q/R278Q*^ (*n* = 9), and *Irf3*^*WT/R278Q*^ (*n* = 6) mice on day 5 after HSV-1 infection, measured by RT-qPCR. **(L–N)***Ifnb, Tnfa*, and *Il6* response 16 h after infection with 1.0 MOI HSV-1 in MBCs treated with 0.5 μM PLX5622 (PLX) for microglia depletion. **(O and P)***Tnfa* and *Il6* response 16 h after infection with 1.0 MOI HSV-1 in MBCs treated with NF-κB inhibitors BMS-345541 (2 μM), PDTC (25 μM), or saline. UI controls were included. **(Q and S)***Ifnb* and *Tnfa* mRNA levels in murine microglia from WT, *Irf3*^*WT/R278Q*^, and *Irf3*^*R278Q/R278Q*^ mice 24 h after HSV-1 infection at increasing virus MOI as indicated. **(R and T)***IFNB1* and *TNFA* mRNA levels in iPSC-derived microglia from the *IRF3*^*WT/R285Q*^ HSE patient and controls 24 h after HSV-1 infection at increasing virus MOI as indicated. Expression data were normalized to β-actin and shown as fold change compared with the UI control. All *in vitro* experiments were performed in triplicates and independently repeated at least three times. Cytokine expression kinetics were compared between the groups using a mixed-effects analysis with Geisser-Greenhouse correction for multiple interacting variables (time and genotype) (A–F), error bars; SEM. Statistical analyses of cytokine and virus levels in brain stem homogenates (G–J and L–T) were analyzed by two-tailed two-way ANOVA for difference of means, followed by an unpaired *t* test of means, error bars; SD. CNS cell experiments (K) were analyzed with a two-tailed one-way ANOVA for difference of means followed by an unpaired *t* test of means. P values <0.05 were considered statistically significant, *P < 0.05, **P < 0.01, and ***P < 0.001. UT, untreated; MOI, multiplicity of infection.

Next, we wanted to examine whether the impaired IFN response observed in *Irf3*^*R278Q/R278Q*^ cells *in vitro* was also seen in the brain *in vivo*. Interestingly, WT and *Irf3*^*R278Q/R278Q*^ mice showed similar expression levels of transcripts for *Ifnb* and the ISG *Mx1* (Fig. 3, A and B). In contrast, proinflammatory cytokines such as *Tnfa*, *Il1b*, *Il6*, and *Ccl2* were significantly elevated in the brain stems of infected *Irf3*^*R278Q/R278Q*^ mice compared with WT (Fig. 3, C–F), starting from the time point of the virus reaching the CNS. This was confirmed by cytokine protein levels in brain stem homogenates from HSV-1–infected mice, including TNFα, CCL2, and IFNγ (Fig. 3, G–J). Of note, we also found elevated *Tnfa* levels in the brain stem of HSV-1–infected heterozygous *Irf3*^*WT/R278Q*^ and *Irf3*^*−/−*^ mice (Fig. 3 K). Examination of serum cytokine levels after HSV-1 infection revealed that a subset of the proinflammatory cytokines was elevated systemically in *Irf3*^*R278Q/R278Q*^ mice (Fig. S3, D–I). To test whether the elevated cytokine expression in the brain of heterozygous and homozygous IRF3 R278Q mice was observed generally in response to inflammatory stimuli, mice were treated with LPS and cytokine transcript levels in whole-brain lysates were examined. While the *Irf3*^*R278Q/R278Q*^ mice had impaired *Ifnb* response to LPS, we did not observe any difference in inflammatory cytokine expression between WT and transgenic mice. This suggests that the observed high levels of cytokine transcripts were specific for the infection (Fig. S3, J–N).

Common for several of the proinflammatory genes elevated in the brains of *Irf3*^*R278Q/R278Q*^ mice after HSV-1 infection is that their transcription is driven by promoters dependent on NF-κB. Therefore, we wanted to examine whether this transcription factor was indeed important for the observed cytokine response *in vitro*. Using mixed brain cell cultures (MBCs) from WT mice we first found that microglia are the predominant cell type responding to HSV-1 with expression of *Ifnb* and NF-κB–driven proinflammatory cytokines *Tnfa*, *Il6*, and *Ccl2*, as gene expression was largely abolished following microglia depletion with the CSFR1 antagonist PLX5622 (Fig. 3, L–N; and Fig. S3, O and P). We next treated MBCs with NF-κB inhibitors BMS-345541 and pyrrolidinedithiocarbamate ammonium (PDTC) or saline control and infected with HSV-1. The MBC model was chosen over *in vivo* treatment to avoid the issue of CNS delivery for the fundamental question of the role of NF-κB in HSV-1–induced cytokine expression. *Tnfa*, *Il6*, and *Ccl2*, three of the transcripts that were elevated in the brain stem of HSV-1–infected *Irf3*^*R278Q/R278Q*^ mice, were induced by HSV-1 infection and all blocked by treatment with BMS-345541, while *Tnfa* and *Il6*, but not *Ccl2*, were blocked by PDTC (Fig. 3, O and P; and Fig. S3 Q). The latter may be explained by *Ccl2* expression being highly responsive to NF-κB and possibly requiring stronger NF-κB inhibition to achieve blockage of expression.

Since *Ifnb* expression in brain stems was unexpectedly similar between WT and *Irf3*^*R278Q/R278Q*^ mice, we hypothesized that the elevated viral load in the *Irf3*^*R278Q/R278Q*^ mice might explain the overall unaltered levels of *Ifnb* despite intrinsic defective responsiveness to infection in the individual cells harboring the IRF3 mutation. To test this, we performed dose-response experiments in murine and human microglia infected with HSV-1 at increasing multiplicity of infection (MOI) *in vitro*. Interestingly, *Irf3*^*R278Q/R278Q*^ microglia required about 100-fold higher HSV-1 inoculation dose than WT microglia to produce similar levels of *Ifnb*, while *Irf3*^*WT/R278Q*^ microglia required about 10-fold higher dose (Fig. 3 Q; and Fig. S3, R and S). This was confirmed in *IRF3*^*WT/R285Q*^ microglia from the HSE patient, where *IFNB* expression at MOI 10 was comparable or slightly elevated relative to healthy donor microglia at MOI 1.0 (Fig. 3 R). Additionally, under conditions giving rise to comparable IFN gene expression in WT and IRF3 mutant cells, *Tnfa*/*TNFA* expression was significantly higher in both murine and human microglia (Fig. 3, S and T).

Collectively, these findings suggest that the elevated viral load in cells with functional IRF3 deficiency leads to enhanced expression of proinflammatory NF-κB–dependent genes and cytokines.

### Increased frequencies of an inflammatory monocyte population in the HSV-1–infected *Irf3*^*R278Q/R278Q*^ mouse brain

**Figure 4.**
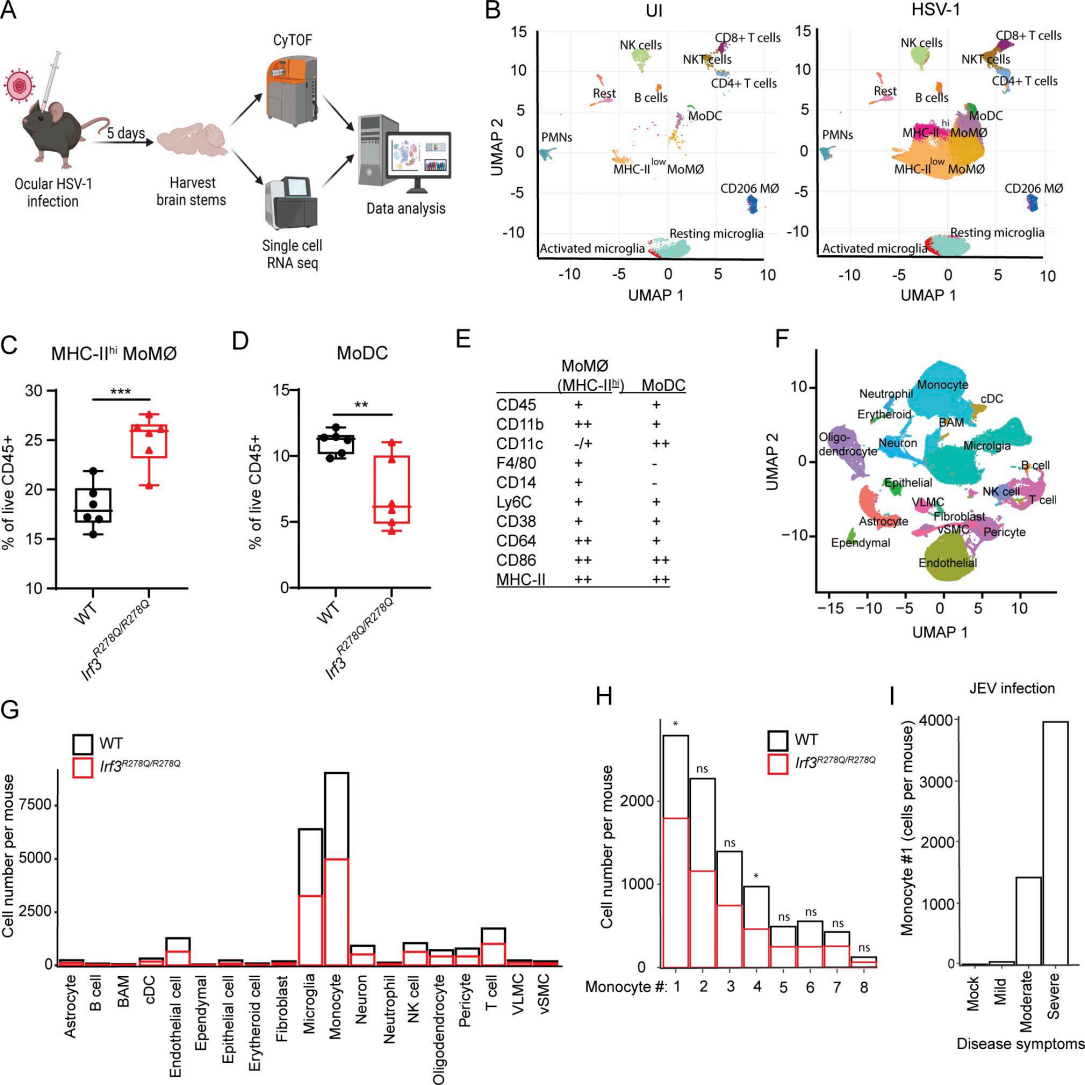
**Increased frequencies of an inflammatory monocyte population in the HSV-1–infected *Irf3*** ^***R278Q/R278Q***^ **brain. (A)** Schematic illustration of single-cell omics pipeline. Mice were infected via the ocular route using 2 × 10^6^ PFU/eye in 5 μl. On day 5 after infection, mice were PBS perfused and brain stems harvested for either CyTOF (WT, *n* = 6; *Irf3*^*R278Q/R278Q*^, *n* = 6; UI, *n* = 6 mice); analysis of CD45^+^ cells or scRNAseq (WT, *n* = 3, each sample contains pools of two to four brain stems; *Irf3*^*R278Q/R278Q*^, *n* = 3, each sample contains pools of two to four brain stems; UI, *n* = 3, each sample contains pools of two brain stems). Image was generated in BioRender. **(B)** UMAP and cell cluster analysis of live CD45^+^ cells acquired by CyTOF from UI or HSV-1–infected brain stems. **(C and D)** Frequency of (C) MHC-II^hi^ MoMØ and (D) MoDC gated as % of live CD45^+^ cells. **(E)** Table of phenotypic markers characterizing the MHC-II^hi^ MoMØ and MoDC cell populations, respectively. The frequencies (C and D) were analyzed with two-tailed Student’s *t* test. Error bars; SD. **(F)** UMAP and cell type annotation of brain stem scRNAseq of WT and *Irf3R*^*278Q/R278Q*^ mice. **(G)** Average cell number of annotated cell types per mouse brain stem of infected WT and *Irf3R*^*278Q/R278Q*^ mice. **(H)** Average cell number of identified monocyte subpopulations per mouse brain stem of infected WT and *Irf3R*^*278Q/R278Q*^ mice. Cell numbers within each subpopulation were first compared across the WT UI compartment using ANOVA test. This was followed by pairwise comparisons between WT and *Irf3R*^*278Q/R278Q*^ mice using Tukey’s honestly significant difference (HSD) test. **(I)** Average number of monocyte #1 population in the brain of mice infected with Japanese encephalitis virus grouped according to severity of disease. P values <0.05 were considered statistically significant, *P < 0.05, **P < 0.01, and ***P < 0.001. MoMø, monocyte-derived macrophage-like cell; MoDC, monocytic-derived dendritic-like cell; VLMC, vascular and leptomeningeal cell; vSMC, vascular smooth muscle cell.

**Figure S4.**
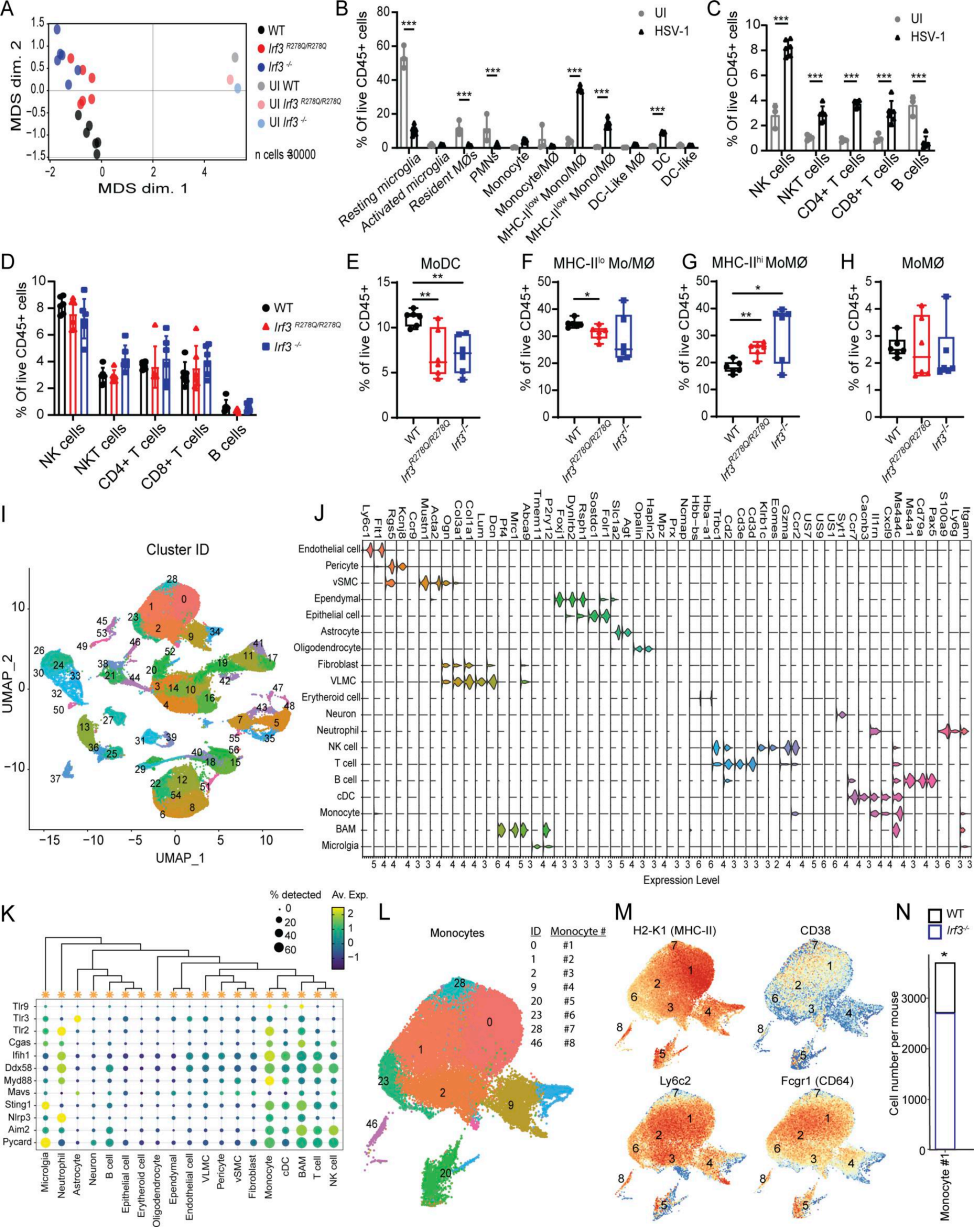
**Recruitment of myeloid cells into the HSV-infected *Irf3*** ^***R278Q/R278Q***^ **brain. (A)** Multidimensional scaling (MDS) of CyTOF samples to visualize the level of similarity of immune infiltration into brain stems of WT, *Irf3*^*R278Q/R278Q*^, *Irf3*^*−/−*^ mice on day 5 after infection. **(B and C)** Immune cell distribution of (B) myeloid and (C) lymphoid cells in brain stems of UI (*n* = 3) and WT HSV-1–infected mice (WT HSV-1, *n* = 6). **(D)** Lymphoid cell distribution in brain stems from WT, *Irf3*^*R278Q/R278Q*^, and *Irf3*^*−/−*^ mice on day 5 after infection. **(E–H)** Myeloid cell distribution in brain stems from WT, *Irf3*^*R278Q/R278Q*^, and *Irf3*^*−/−*^ mice on day 5 after infection. **(B–H)** Data are represented as % of live CD45^+^ cells and analyzed by two-tailed two-way ANOVA for difference of means, followed by two-tailed unpaired *t* test of means, error bars; SD. P values <0.05 were considered statistically significant. **(I)** UMAP and cluster identification of scRNAseq data. **(J)** Marker table used for annotation of cell types. **(K)** Dot plot for expression of genes encoding sensors and adaptor molecules related to innate immune sensing of HSV-1 in annotated cell types. **(L)** Subclustering analysis of monocytes. **(M)** Mapping of transcript levels for four markers used to identify MHC-II^hi^ MoMØ onto the monocyte subpopulations identified by scRNAseq. **(N)** Monocyte #1 cell number per mouse in WT and *Irf3*^*−/−*^ mice from scRNAseq. P values below 0.05 were considered significant, *P < 0.05, **P < 0.01, and ***P < 0.001.

Based on the identified elevated expression of a panel of inflammatory cytokines, we were interested in examining whether the HSV-1–infected mouse brain also exhibited differential distribution of immune cell populations between WT and *Irf3*^*R278Q/R278Q*^ mice. To this end, we isolated cells from the brain stem and performed multiplex suspension mass cytometry (cytometry by time of flight [CyTOF]) and single-cell RNA sequencing (scRNAseq) (Fig. 4 A and Fig. S4 A). The mass cytometry panel covered extracellular cell type and activation markers (Table S1). We first characterized the immune cell profile in WT mice after infection. In agreement with previous reports (Conrady et al., 2013; Katzilieris-Petras et al., 2022; Menasria et al., 2015), we observed strong recruitment of several immune cell types, with monocytes being by far the most abundant (Fig. 4 B; and Fig. S4, B and C). When comparing WT and *Irf3*^*R278Q/R278Q*^ mice, we observed no difference in the abundance of the lymphoid cells (Fig. S4 D). This was also the case when extending the comparison to fully IRF3-deficient mice (Fig. S4 D). By contrast, within the myeloid cell compartment, we identified two populations that showed differential abundance in *Irf3*^*R278Q/R278Q*^ mice compared with WT mice (Fig. 4, C and D; and Fig. S4, E–H). One of these populations exhibited high expression of MHC-II and other activation markers (MHC-II^hi^ monocyte-derived macrophage [MoMØ]), and the frequency of this population was elevated in *Irf3*^*R278Q/R278Q*^ mice (Fig. 4, C and E). The other population showed a profile in-between monocytes and dendritic cells (DCs), indicative of a monocyte-derived DC, and the frequency was decreased in *Irf3*^*R278Q/R278Q*^ mice compared with WT (Fig. 4, D and E). These two myeloid cell populations showed the same pattern of abundance in the brain stem of infected *Irf3*^*−/−*^ mice (Fig. S4, E and G).

Analysis of the scRNAseq data enabled us to identify all relevant brain-resident and -recruited immune cells (Fig. 4 F and Fig. S4, I and J). Confirming our findings from CyTOF, we identified a significant increase in immune cells, and the most abundant cells in absolute counts per mouse were the infiltrating monocytes (Fig. 4 G). In addition, monocytes and neutrophils showed the highest expression of innate immune sensors related to HSV-1 infections (Fig. S4 K). By subcluster analysis of the monocyte population, we identified 8 clusters (Fig. S4 L). Among these populations, #1 and #4 showed differential abundance between WT and *Irf3*^*R278Q/R278Q*^ mice, with higher abundance of #1 and lower for #4 in *Irf3*^*R278Q/R278Q*^ compared with WT (Fig. 4 H). Closer examination of the markers identified to be expressed in the MHC-II^hi^ MoMØ population revealed a strong overlap with the monocyte population #1 (Fig. S4 M). This population #1 was also enriched in the brain stem of HSV-1–infected *Irf3*^*−/−*^ mice (Fig. S4 N). To examine whether monocyte population #1 may be a general feature of high viral load in the brain, we analyzed a scRNAseq dataset from mice infected with Japanese encephalitis virus (Yang et al., 2024). Interestingly, monocyte #1 was indeed identified in the brains of infected mice, and the abundance correlated with disease severity (Fig. 4 I). Collectively, these data suggest that HSV-1 brain infection of *Irf3*^*R278Q/R278Q*^ mice leads to differential regulation of two recruited myeloid cells, one of which shows higher abundance, and a profile of sensing HSV-1.

### Enriched inflammatory monocyte population exhibits a phenotype associated with brain pathology

**Figure 5.**
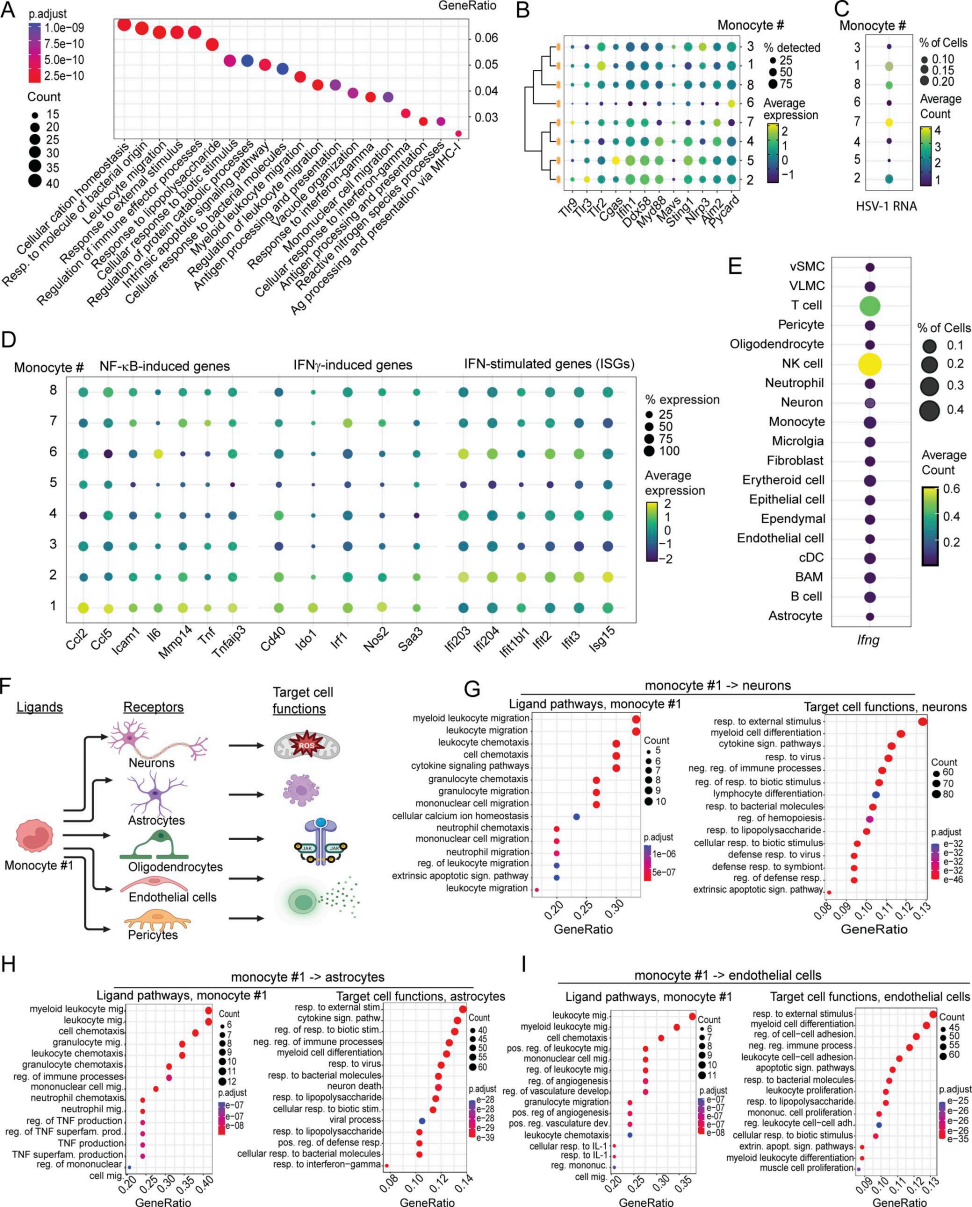
**Characterization of an inflammatory monocyte population enriched in *Irf3*** ^***R278Q/R278Q***^ **mice. (A)** Dot plot showing enriched GO terms in monocyte #1. **(B–D)** Dot plots showing expression in monocyte subpopulations of (B) transcripts for proteins involved in HSV-1 sensing; (C) HSV-1 RNA; and (D) selected transcripts for NF-κB–stimulated genes, IFNγ-induced genes, and ISGs. **(E)** Dot plot showing expression of *Ifng* across cell types annotated in the scRNAseq data set. **(F)** Illustration of setup for analysis of cell–cell communication between monocyte #1 and brain-resident cells using NicheNet. Image was generated in BioRender. **(G–I)** Dot plot showing enriched GO terms at the level of ligands and target cell functions from the NicheNet analysis of the communication between monocyte #1 and (G) neurons, (H) astrocytes, and (I) endothelial cells. Expression levels (B and D) are shown as average expression, and average count of expressing cells (C and E) and range color coded from blue (lowest) to yellow (highest). The hypergeometric distribution was employed to calculate P values for GO enrichment analysis. The false discovery rate (FDR) was subsequently applied to correct for multiple testing. Adjusted P value shown in color range (red [lowest] to blue [highest]). P values <0.05 were considered statistically significant.

**Figure S5.**
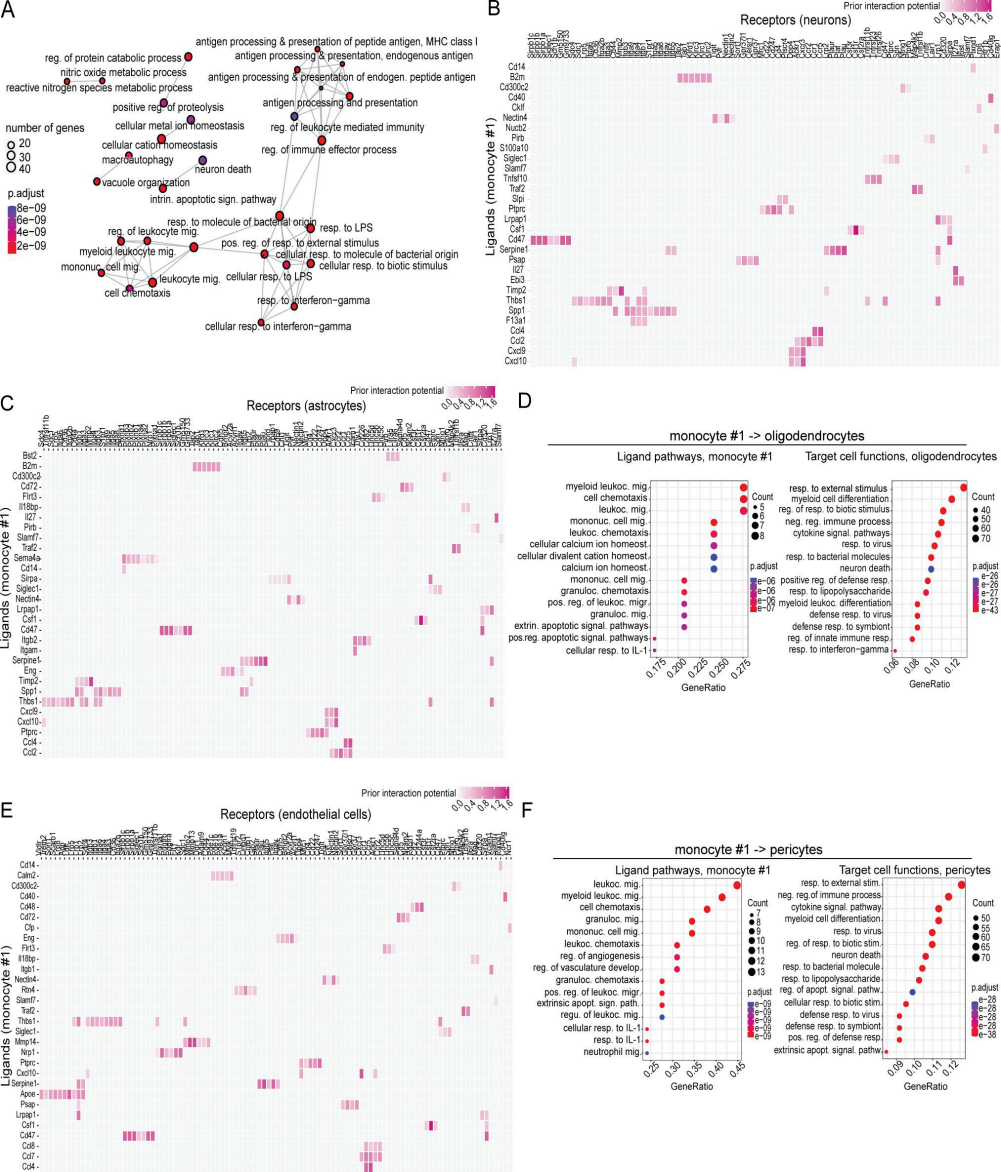
**Characterization of inflammatory monocyte population enriched in *Irf3*** ^***R278Q/R278Q***^ **mice. (A)** Network plot of the enriched GO terms in monocyte #1. **(B)** Top ranking ligand–receptor pairs in the NicheNet analysis of communication between monocyte #1 and neurons. **(C)** Top ranking ligand–receptor pairs in the NicheNet analysis of communication between monocyte #1 and astrocytes. **(D)** Dot plot showing enriched GO terms at the level of ligands and target cell functions from the NicheNet analysis of the communication between monocyte #1 and oligodendrocytes. **(E)** Top ranking ligand–receptor pairs in the NicheNet analysis of communication between monocyte #1 and endothelial cells. **(F)** Dot plot showing enriched GO terms at the level of ligands and target cell functions from the NicheNet analysis of the communication between monocyte #1 and pericytes. The hypergeometric distribution was employed to calculate P values for GO enrichment analysis. The FDR correction was subsequently applied to correct for multiple testing. Adjusted P values are shown using a color range from red (lowest) to blue (highest). P values below 0.05 were considered significant, *P < 0.05, **P < 0.01, and ***P < 0.001. FDR, false discovery rate.

To gain insight into the myeloid cell population showing differential abundance in the brain stem of HSV-1–infected WT and *Irf3*^*R278Q/R278Q*^ mice, we further analyzed our scRNAseq dataset. First, we performed pathway enrichment analysis for monocyte #1. The transcriptome of monocyte #1 was enriched for pathways related to leukocyte migration, response to bacteria, and antigen presentation (Fig. 5 A and Fig. S5 A). To our surprise, we found no enrichment of processes related to virus infections. When comparing the monocyte subpopulations for expression of transcripts encoding PRRs and signaling adaptors involved in sensing and signaling to HSV-1 infection, we noted that monocyte #1 had the highest expression of *Tlr2*, but not other HSV-1–sensing PRRs (Fig. 5 B). When correlating this with abundance of viral transcripts in the monocytes, we found that monocyte #1 contained second most viral transcripts, only surpassed by monocyte #7, which generally expressed low levels of the sensor-signaling genes (Fig. 5 C). The analyses of enriched pathways, PRR gene expression, and viral transcripts suggested that monocyte #1 responded to the sensing of HSV-1 with activation of a program resembling bacterial infections. Since the NF-κB pathway is a prominent component of such programs, we compared the expression of NF-κB–stimulated genes, IFNγ-induced genes, and ISGs between the monocyte subpopulations. Interestingly, monocyte #1 expressed the highest levels of NF-κB–stimulated genes and IFNγ-induced genes but did not express high levels of ISGs (Fig. 5 D). The population showing the highest levels of ISGs was monocyte #2, which had high expression of *Tlr3*. Defects in the TLR3 pathway are associated with development of HSE in humans (Zhang et al., 2007). The IFNγ response, which was elevated in the infected *Irf3*^*R278Q/R278Q*^ brain (Fig. 3 I), was derived primarily from NK cells at the time point examined (Fig. 5 E).

To examine how monocyte #1 may influence brain structure and function and the development of HSE, we used NicheNet to infer communication pathways and functions to brain cells focusing on neurons, astrocytes, oligodendrocytes, endothelial cells, and pericytes (Fig. 5 F). The pathways inferred to be engaged by monocyte #1 to signal to neurons included cytokines and extrinsic apoptotic signaling pathways (Fig. 5 G). The analysis also yielded pathways related to leukocyte chemotaxis, which seem less likely to be physiologically relevant in this context. The neurons did respond to the inferred monocyte #1 signals as indicated by the general enrichment of responses to external stimuli. Notably responses to cytokine signaling, response to bacteria and viruses, likely reflecting PRR signaling, and extrinsic apoptotic signaling pathways were enriched. Specifically, monocyte #1 was found to promote communication through the TNF superfamily member (TNFSF)10/TRAIL pathway (Fig. S5 B), known to drive neuronal cell death under inflammatory conditions (Aktas et al., 2005). For the monocyte #1–astrocytes communication, we noted that monocyte #1–derived TNF and the TNFSF pathways were enriched. The astrocytes correspondingly instigated an elevated cytokine signaling response, thus activating defense activities and programmed cell death (Fig. 5 H). Notably, signaling to the TNFα receptor subunit *Tnf1sf1b* on astrocytes was highlighted (Fig. S5 C). Oligodendrocytes were also highly responsive to the cytokine signals sent from monocyte #1 and, like the astrocytes, were enriched for processes associated with neuronal cell death (Fig. S5 D). Given the observed breakdown of the BBB (Fig. 1, O and P), we were also interested in the possible interaction between monocyte #1 and associated cell types. The pathways enriched in the monocyte #1 ligands involved in cross talk with endothelial cells included numerous terms involved in vasculature and specifically the IL-1 pathway (Fig. 5 I). The endothelial cells, on their side, were activated by the signals sent by monocyte #1 to support leukocyte infiltration and upregulated signals for apoptosis. Specific receptors mediating the monocyte #1–derived response in the endothelial cells included *Tnf1sf1b* and *IL6ra* (Fig. S5 E). The enrichment of monocyte #1–derived signals for apoptosis for BBB-associated cells was also seen in pericytes (Fig. S5 F). Thus, monocyte #1 is an inflammatory monocyte population exposed to high levels of virus and IFNγ in the HSV-1–infected brain to drive a transcriptional program characterized by expression of NF-κB– and IFNγ-stimulated genes. This population engages in extensive cross talk with brain cells, promoting processes associated with pathologies.

### Blockage of NF-κB mitigates disease severity of HSV-1–infected IRF3 R278Q mice

**Figure 6.**
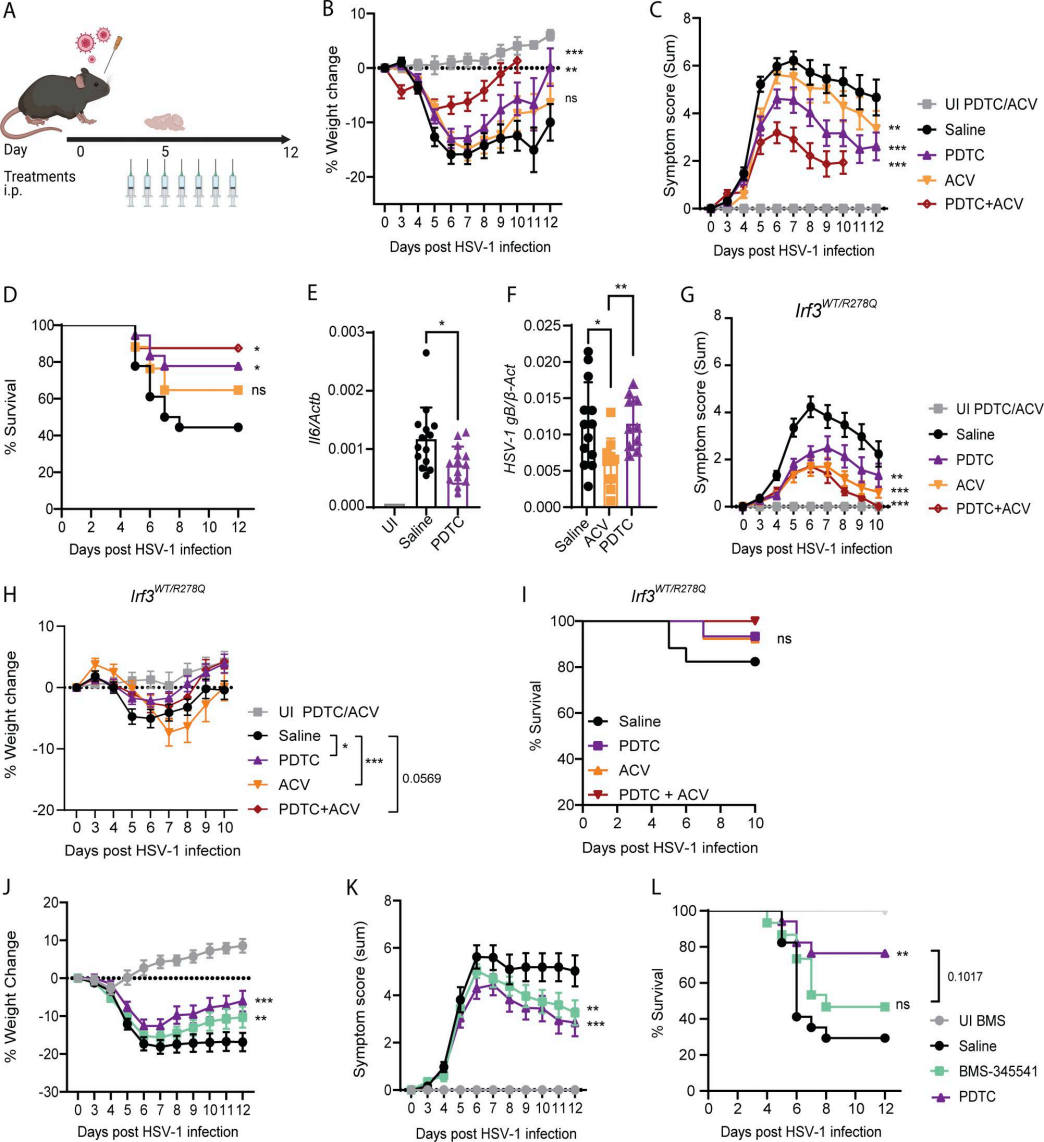
**Blockage of NF-κB activation mitigates disease severity of HSV-1–infected IRF3 R278Q mice. (A)** Mice were infected via the ocular route using 2 × 10^6^ PFU/eye in 5 μl. Treatments were initiated on day 3 after infection and administered by i.p. injection once daily until tissue harvest on day 5 or for 7 consecutive days for therapeutic intent. **(B–D)***Irf3*^*R278Q/R278Q*^ mice (littermates) were infected with HSV-1 and randomized to treatment with 50 mg/kg ACV (*n* = 17), 10 mg/kg PDTC, (*n* = 18), ACV + PDTC (*n* = 16), or saline (*n* = 18). *n* = 4 UI controls treated with ACV or PDTC were included. Disease development was followed as % weight change (B) and symptom score (C) until mice reached humane endpoint or full recovery of 100% of starting body weight, represented in (D) survival curve. **(E and F)** Brain stems from *Irf3*^*R278Q/R278Q*^ mice treated with saline (*n* = 13–14), PDTC (*n* = 10–14), or ACV (*n* = 10) were harvested 5 days after infection with HSV-1 and *Il6* expression (E), and *HSV-1 gB* transcripts (F) were analyzed by RT-qPCR and normalized to β-actin (*Actb*). UI controls were included (*n* = 3). **(G–I)***Irf3*^*WT/R278Q*^ heterozygote littermates were infected with HSV-1 and randomized to treatment with 50 mg/kg ACV (*n* = 13), 10 mg/kg PDTC (*n* = 15), ACV + PDTC (*n* = 13), or saline (*n* = 17). UI controls treated with ACV (*n* = 4) or PDTC (*n* = 4) were included. **(G)** Symptom score. **(H)** % Weight change. **(I)** Survival curve. **(J–L)***Irf3*^*R278Q/R278Q*^ mice (littermates) were infected with HSV-1 and randomized to treatment with 25 mg/kg BMS-345541 (*n* = 15), 10 mg/kg PDTC, (*n* = 17), or saline (*n* = 17). UI controls (*n* = 4) treated with BMS-345541 were included. Disease development was followed as % weight change (J) and symptom score (K) until mice reached humane endpoint or full recovery of 100% of starting body weight, represented in (L) survival curve. Treatment experiments were independently repeated two times with similar results. Disease development (weight change and symptom score) was compared between the groups using a mixed-effects analysis with Geisser-Greenhouse correction for multiple interacting variables (time and genotype). Error bars; SEM. Survival was analyzed using log-rank Mantel–Cox test. Dead animals were censored in the graphs and thus represented in the graphs with weight and symptom score at time of death. Statistical analyses of gene expression in brain stems (E and F) were analyzed by two-tailed one-way ANOVA for difference of means, followed by an unpaired *t* test of means, error bars; SD. P values <0.05 were considered statistically significant, *P < 0.05, **P < 0.01, and ***P < 0.001.

Based on the observed elevation of inflammatory responses dependent on NF-κB in *Irf3*^*R278Q/R278Q*^ mice, we hypothesized that HSE disease symptoms and death in IRF3 R278Q mice is dependent on activity of this transcription factor. To test this, we treated mice with the NF-κB inhibitor PDTC, which is reported to be BBB penetrating (Kan et al., 2016; Nurmi et al., 2004), alone or in combination with the antiviral nucleoside analog acyclovir (ACV). The treatment was initiated on day 3 after infection, i.e., after the virus has reached the CNS, and hence mimicking a treatment situation (Fig. 6 A). Importantly, *Irf3*^*R278Q/R278Q*^ mice treated with PDTC showed a milder course of disease, with significantly less weight loss, symptom scores, and risk of death, compared with infected animals not receiving the NF-κB inhibitor (Fig. 6, B–D). Of note, PDTC treatment reduced levels of the NF-κB–dependent *Il6* transcripts (Fig. 6 E) but did not affect the viral load in brain stems from HSV-1–infected *Irf3*^*R278Q/R278Q*^ mice (Fig. 6 F). Treatment of *Irf3*^*R278Q/R278Q*^ mice with ACV alone from day 3 after infection failed to significantly ameliorate disease development, although it did reduce viral load (Fig. 6, B–D and F). In combination with PDTC, ACV treatment led to further improvement of the disease outcome (Fig. 6, B–D). To fully mimic the HSE patient, we also treated heterozygous *Irf3*^*WT/R278Q*^ mice with PDTC. As for the homozygous mice, we observed significantly improved disease outcome measured by symptom score (Fig. 6 G) and weight loss (Fig. 6 H) but did not reach significance for survival (Fig. 6 I). In addition, the combination of PDTC and ACV almost completely eliminated symptom development in the heterozygous *Irf3*^*WT/R278Q*^ mice (Fig. 6 G).

PDTC was selected for blockade of NF-κB because of its ability to cross the BBB (Kan et al., 2016; Nurmi et al., 2004). However, PDTC is not a selective NF-κB inhibitor (Lee et al., 2017; Liddell et al., 2016), and we therefore also tested the highly selective IκB kinase (IKK) inhibitor BMS-345541 (Burke et al., 2003). Although this compound does not cross efficiently into the brain (Herrmann et al., 2005), we reasoned that it might passively diffuse into the lesions, as we have observed breakdown of the BBB during HSE-like disease (Fig. 1, O and P). Indeed, treatment of *Irf3*^*R278Q/R278Q*^ mice with BMS-345541 from day 3 after infection significantly improved symptom scores and weight loss but did not improve survival significantly (Fig. 6, J–L).

Taken together, our data suggest that HSE-like disease development in heterozygous and homozygous IRF3 R278Q mice is at least in part driven by excessive activation of NF-κB breakdown dependent response downstream of elevated viral load.

## Discussion

Human genetics studies of HSE have revealed that defects in the type I IFN pathways predisposes to this disease (Andersen et al., 2015; Audry et al., 2011; Bastard et al., 2021; Bucciol et al., 2023; Casrouge et al., 2006; Dupuis et al., 2003; Herman et al., 2012; Pérez de Diego et al., 2010; Sancho-Shimizu et al., 2011; Zhang et al., 2007). Cell culture data from iPSC-derived neurons, oligodendrocytes, and astrocytes from HSE patients have suggested that the genetic defects lead to cell-intrinsic inability to control viral replication in the brain (Gao et al., 2021; Lafaille et al., 2012), potentially arguing for the pathogenesis to be driven mainly by viral cytopathic effects. However, the mechanistic basis for development of HSE in susceptible individuals remains incompletely understood. Here we report that mice carrying the IRF3 R278Q amino acid substitution, corresponding to the human HSE-associated IRF3 R285Q gene variant (Andersen et al., 2015), exhibit impaired antiviral control in the both the peripheral tissue and CNS, leading to higher viral inoculum into the CNS, where viral replication is further enhanced. This leads to elevated expression of inflammatory chemokines and cytokines through the NF-κB pathway in microglia, although the overall type I IFN response is not significantly reduced. This, in turn, directs infiltration of inflammatory monocytes, which become activated into disease associated subsets, thus driving an immunopathological response dependent on signaling through the NF-κB pathway. Our results suggest that HSE is a disease of HSV-1–triggered immune dysfunction driven by a combination of lack of control of viral replication and hyper-inflammatory response to elevated and sustained viral presence in the CNS.

The type I IFN system is essential for prevention of HSE and for control of HSV-1 infection in the brain in the model used in this study (Bastard et al., 2021; Reinert et al., 2021). We previously reported that microglia are responsible for the bulk of the first wave of type I IFN and inflammatory cytokines after HSV-1 enter the CNS (Katzilieris-Petras et al., 2022; Reinert et al., 2016). Accordingly, depletion of microglia rendered mice highly susceptible to HSV-1 infection (Katzilieris-Petras et al., 2022), and iPSC-derived microglia exposed to HSV-2 can protect neurons from infection in a type I IFN–dependent manner (Reyahi et al., 2023). In line with this, other studies have also found microglia to be essential for defense against acute challenge with a mouse coronavirus or West Nile encephalitis virus (Funk and Klein, 2019; Wheeler et al., 2018). When following the cytokine response beyond the first few days of infection, we reported that microglia were not the main cytokine source from day 3 and onward, correlating with influx of myeloid cells. In the current study, we observed elevated expression of inflammatory cytokines in *Irf3*^*R278Q/R278Q*^ mice early after viral entry into the CNS and unaltered expression of *Ifnb*. The cytokine and chemokine response to HSV-1 from brain-resident cells from *Irf3*^*R278Q/R278Q*^ mice was mainly from microglia. Importantly, upon treatment of microglia from the patient with IRF3 R285Q HSE gene variant or from *Irf3*^*R278Q/R278Q*^ mice with increasing amounts of HSV-1, we observed that increasing doses of virus enabled significant *IFNB/Ifnb* expression and led to augmented expression of *TNFA*/*Tnfa* transcripts. This suggests a dual role for microglia in the immune response to HSV-1 in the CNS. First, instigation of defense activity through type I IFNs, which exert their action on neurons and astrocytes (Fruhwurth et al., 2023; Hayes et al., 2022; Rosato and Leib, 2015). Second, recruitment of immune cells from the periphery, with monocytes constituting the majority. This set of cells also contributes to host defense, although the mechanism is largely unknown (Fruhwurth et al., 2023). In individuals with a functional type I IFN system, this allows induction of an antiviral state broadly around the infection foci and clearance of the virus, thus leading to modest pathology. However, if type I IFN immunity is impaired, e.g., due to reduced IRF3 function, the antiviral state is not induced broadly around the infected cells, consequently leading to elevated viral load. This in turn leads to elevated PRR engagement and disproportionately high expression of inflammatory cytokines/chemokines *versus* type I IFNs, hence promoting inflammation and immunopathology over direct antiviral activity. We found that inhibition of NF-κB signaling on day 3 and onwards after infection reduced pathology and disease development.

When profiling immune cells in the brain stem of HSV-1–infected WT vs *Irf3*^*R278Q/R278Q*^ mice, we found a larger fraction of infiltrating monocytes in the *Irf3*^*R278Q/R278Q*^ mice. Consistently, we identified higher levels of CD45^+^ cells in the infected areas of the brain stem in *Irf3*^*R278Q/R278Q*^ mice. Using multiplex suspension mass cytometry and scRNAseq, we identified two monocyte populations with differential abundance in WT and *Irf3*^*R278Q/R278Q*^ mice. Notably, one of these subpopulations, monocyte #1, belonging within the class of Ly6C^+^ inflammatory monocytes (Yang et al., 2014), was more abundant in *Irf3*^*R278Q/R278Q*^ mice. Although containing high levels of viral transcripts, the transcriptome of monocyte #1 was not enriched for pathways related to responses to virus infections. By contrast, the transcriptome profile of monocyte #1 was enriched for terms related to response to bacterial infections, response to IFNγ, and antigen presentation. Consistently, monocyte #1 showed high expression of TLR2, which potently induces NF-κB–activated genes (Takeuchi and Akira, 2010), and this class of genes was indeed highly upregulated in monocyte #1 cell transcripts. This was accompanied by upregulation of a large panel of IFNγ-induced genes, many of which are induced synergistically with NF-κB (Paludan, 2000). NicheNet-based cell–cell communication analyses revealed that the IFNγ signal to monocyte #1 was received from NK and T cells and suggested that monocyte #1 communicated extensively to neurons, astrocytes, oligodendrocytes, and endothelial cells, e.g., through TNFα and other TNFSF pathways. We propose that the virus- and cytokine-rich milieu in the HSV-1–infected *Irf3*^*R278Q/R278Q*^ mouse brains promote the development and activation of the monocyte #1 subpopulation, which contributes to disease pathology. This population may in fact be general for viral encephalitis, as we also identified monocyte #1 in mice infected with Japanese encephalitis virus correlating with disease severity (Yang et al., 2024). Of note, the HSE patient carrying the IRF3 R285Q mutation had pleocytosis in the cerebrospinal fluid dominated by mononuclear cells (Andersen et al., 2015), indicating presence of activated monocytes similar to what was found in the mouse model.

An important finding of the present work is the observation that treatment with inhibitors of NF-κB ameliorated disease outcome, without affecting the viral load. The therapeutic effect of NF-κB inhibition was even more pronounced if combined with antiviral therapy. In our experiments, we initiated treatment at a time point after the virus had reached the brain, thus mimicking a therapeutic setting. The inhibition of NF-κB activity led to reduced levels of inflammatory gene expression in the brain stem and reduced cell death in the infected area. This suggests that the late NF-κB–driven response in the infected brain promotes inflammatory cell death, which may accelerate disease progression. A study of human brain organoids infected with HSV-1 has also reported an effect of blockage of cell death modalities on brain cell survival in combination with antiviral therapy (Rybak-Wolf et al., 2023). Our study does not exclude possible beneficial roles for NF-κB in brain host defense against HSV-1, particularly in the early phase of infection, since we initiated treatment on day 3 of infection. We used two different inhibitors of NF-κB, namely, PDTC and BMS-345541. While both inhibitors are well described to block NF-κB activation, they also have limitations. For instance, PDTC is not specific for NF-κB (Lee et al., 2017; Liddell et al., 2016) but was chosen because it has been demonstrated to cross the BBB and work in the brain (Kan et al., 2016; Nurmi et al., 2004). By contrast, although BMS-345541 is rather specific for the IKK complex (Burke et al., 2003), available data suggest that it does not efficiently cross the BBB (Herrmann et al., 2005). However, since the BBB was disrupted in the HSV-1–infected *Irf3*^*R278Q/R278Q*^ mice, mainly driven by inflammation (Ding et al., 2025), we found that there was an evidence-based rationale for using this compound for treatment. Future studies should test a broader panel of mice, e.g., *Ifnar1*^*−/−*^ and *cGas*^*−/−*^ mice (Reinert et al., 2021), to explore whether pathological activation of NF-κB is broadly observed during HSV-1 brain infection in the context of defective IFN immunity. The results from this study suggest that specific and BBB-permeable NF-κB inhibitors in combination with antiviral therapy have potential for treatment of HSE.

Previous clinical studies have failed to demonstrate a beneficial effect of the treatment of HSE patients with glucocorticoids in combination with ACV (Hodzic et al., 2023). This may raise some concern as to the potential for treatments targeting the pathways promoting immunopathology. However, it should be highlighted that glucocorticoids block numerous immune pathway, including the action of type I IFN (Flammer et al., 2010) and Th1 responses (Franchimont et al., 2000), thus impairing the endogenous beneficial antiviral response. Moreover, glucocorticoids have multiple effects on brain activities that are already affected in the HSV-1–infected brain (Gonzalez Dosal et al., 2011; Kavouras et al., 2007; Ren et al., 2023; Wozniak et al., 2009). This includes neuronal mitochondrial function, programmed cell death, and tau pathology (Behl et al., 1997; Du et al., 2023; Suwanjang et al., 2019). Therefore, glucocorticoids may amplify a panel of neuropathological activities induced during HSV-1 infection while blocking beneficial antiviral activities. We were not able to do a direct comparison between dexamethasone and NF-κB activation inhibitors in the HSV-1 brain infection model, since the rapid loss of lean body mass in mice receiving dexamethasone treatment did not allow additional HSV-1 infection due to ethical considerations (Wang et al., 2023). Based on the data from the present study, we propose that an approach selectively targeting the pathways known to contribute to HSE pathogenesis should be explored in future development of HSE therapies.

Altogether, we report that impaired IRF3 signaling results in enhanced HSV-1 entry into CNS following mucosal infection and subsequent viral replication in the CNS. The high viral load leads to strong activation of microglia and elevated expression of genes driven by the NF-κB pathway, while the type I IFN pathway shows same net output in IRF3-deficient microglia as WT microglia. In the context of reduced IRF3 activity, inflammatory monocytes are recruited into the CNS, where they develop into hyperinflammatory cell subsets and promote brain pathology. Our work provides significant new understanding of the pathophysiological mechanisms driving this devastating disease. Importantly it highlights that curbing these pathological inflammatory mechanisms, while leaving the antiviral mechanisms untouched, represents a promising therapeutic option for HSE treatment in combination with antiviral therapy. It is also noteworthy that such treatment could potentially reduce some of the sequelae frequently observed in patients that survive HSE, given the known prolonged immune activity in the postinfectious brain. As a more general comment, we propose that combining human genetics with mouse models that genocopy identified pathogenic human variants and stem cell–based human model systems represent a powerful toolbox to advance the understanding of complex human diseases and narrowing the translational gap in experimental medicine.

## Materials and methods

### Study approval

The National Committee on Health Research Ethics and the Danish Data Protection Agency approved the study (project #1–10-72-586-12). Written consent was obtained from the patient prior to inclusion. All personal information is protected as required according to the Data Protection Agency and relevant Danish laws.

All animal studies were approved by the Animal Ethics Committee at the Danish Veterinary and Food Administration (permission numbers 2016-15-0201-01085, 2021-15-0201-01084, 2016-15-0201-01072, and 2021-15-0201-01087). All experiments were carried out in accordance with local regulations, Danish Animal Welfare Act for the Care and Use of Animals for Scientific Purposes. All animal experiments were designed and carried out following ARRIVE guidelines.

### iPSC clone reprogramming from human IRF3 R285Q patient fibroblasts

Fibroblast (P1) generated from a skin biopsy from a 15-year-old adolescent with previous HSE (Andersen et al., 2015) were cultured in DMEM (4.5 g/l glycose; 10443142; HyClone) supplemented with 10% FCS (SV310601; HyClone), 1% L-Glutamine (PAA M11-004), and 1% NEAA (11140050; Thermo Fisher Scientific). Medium was changed every 2–3 days, and the cells were passaged with trypsin once before reprogramming.

The day before reprogramming (day −1), fibroblasts were plated at 1 × 10^4^ cells/cm^2^ in Matrigel (354277; Corning)-coated 35-mm cell culture dishes in fibroblast culture medium. Fibroblasts were reprogrammed using the ReproRNATM-OKSGM kit (05930; STEMCELL Technologies) according to the manufacturer’s instructions. Briefly, cells were transfected with Repro-RNA cocktail in fibroblast medium (STEMCELL Technologies). 1 day after transfection, the medium was changed to growth medium composed of advanced DMEM (12491015; Thermo Fisher Scientific), 10% FBS (SV310601; HyClone), 1% L-Glutamin (PAA M11-004), recombinant B18R protein, and 1.2 µg/ml pyromycin (A1113803; Thermo Fisher Scientific). Medium was changed daily for 5 days. Thereafter, pyromycin was withdrawn from the medium for another 2 days. On day 8 after transfection, medium was exchanged for ReproTeSR medium (STEMCELL Technologies) supplemented with B18R, and medium was changed daily until day 14 after transfection. Thereafter, B18R was withdrawn from the ReproTeSR medium. Medium was changed daily until iPSC clones appeared and grew large enough for manual selection, between days 20 and 25.

iPSC clones were picked manually, and each clone was seeded separately in Matrigel-coated 35-mm cell culture dishes in mTeSR1 medium (STEMCELL Technologies). Medium was replaced every day, and colonies were first passaged manually to a new dish when growing too large and thereafter passaged 1:2 with EDTA (Thermo Fisher Scientific) when reaching 70–80% confluency. Five separate picked clones reached high enough quality for cryopreservation. iPSCs from clones IRF3iC4 and IRF3iC6 were harvested at passage 6 and processed for G-banding karyotyping. No large chromosomal abnormalities were observed (Giemsa-banding karyotyping, data not shown). FACS analysis showed that iPSC had no expression of SSEA-1 (100% negative) and almost all cells express SSEA-4 and OCT4 (over 98% positive cells for both clones, data not shown), indicating that the clones contain pure iPSCs expressing pluripotency markers. This was further confirmed by staining for the pluripotency markers NANOG and OCT4 (Fig. S1, A and D). Differentiation of the iPSCs into the three germ layers using the Trilineage differentiation kit showed that iPSCs were able to differentiate toward endodermal, ectodermal, and mesodermal cells (data not shown).

Three human control iPSC lines WTSIi015-A (EBiSC; Sigma-Aldrich), BION-C (EBiSC; Sigma-Aldrich), H9 (WA-09; WiCell), and ChiPSC22 (Takara Bio) were used. WTSIi015-A was used as a control for IRF3-C6 microglia, and BION-C and H9 were used as a control for IRF3-C6 neurons, and ChiPSC22 was used as a control for IRF3-C6 astrocytes. Prior to selection for experiments, control lines and patient clones were compared on select project-relevant readouts (HSV-1–induced *IFNB* expression for microglia and HSV-1 replication for neurons; Fig. S1, I and J), and a representative control line was selected. iPSCs were maintained on Matrigel (Corning) in mTeSR1^+^ medium (STEMCELL Technologies).

### Generation of human iPSC-derived microglia, neurons and astrocytes

Microglia and neurons were generated as previously published (Fruhwurth et al., 2023). In short, microglia was generated as follows: iPSC colonies were dissociated into single cells using TrypLE Express (Thermo Fisher Scientific). 4 × 10^6^ iPSCs were seeded in 2 ml embryonic body (EB) medium (mTeSR1^+^ medium supplemented with 10 μM ROCK inhibitor, 50 ng/ml BMP-4, 20 ng/ml SCF, and 50 ng/ml VEGF-121 [PeproTech]) in AggreWell 800 24-well plate (STEMCELL Technologies). Cells were cultured for 4 days to form EBs. EBs were harvested using an inverted cell strainer (40 μm), and ∼15 EBs were seeded in 6-well plates in hematopoietic medium (X-VIVO 15 medium [Lonza] supplemented with 2 mM Glutamax [Gibco], 100 U/ml penicillin and 100 μg/ml streptomycin [Gibco], 55 μM β-mercaptoethanol [Gibco], 100 ng/ml human M-CSF [PeproTech], and 25 ng/ml human IL-3 [PeproTech]). Primitive macrophage precursors were harvested on day 30 and plated at a density of 6 × 10^4^ cells/cm^2^ in microglia medium (MiM, Advanced DMEM F12 medium [Gibco] supplemented with 2 mM Glutamax, 100 U/ml penicillin, 100 μg/ml streptomycin, 55 μM β-mercaptoethanol, 100 ng/ml human IL-34 [PeproTech], and 10 ng/ml human GM-CSF). Microglia were differentiated in MiM for 6–9 days with full medium change every other day.

Neurons were generated as follows: 1 day prior to neuronal induction, iPSC were passaged using EDTA (Thermo Fisher Scientific). Upon 100% confluency the following day, medium was switched to neural maintenance medium (NMM, DMEM/F12 and neurobasal medium [1:1] supplemented with 1× N2 supplement, 1× B27 supplement, 50 µM 2-mercaptoethanol, 0.5× nonessential amino acids, 100 μM L-glutamine [all from Life Technologies], 2,500 U/ml penicillin/streptomycin [GE Healthcare], 10 µg/ml insulin, and 0.5 mM sodium pyruvate [Sigma-Aldrich]). NMM was further supplemented with 500 ng/ml mouse Noggin/CF chimera (R&D Systems) and 10 μM SB431542 (STEMCELL Technologies). The cells were maintained in this medium for 10–12 days. The obtained neuroepithelial sheet was dissociated using 10 mg/ml Dispase II (Thermo Fisher Scientific) and seeded on laminin-coated plates (1–2 µg/cm^2^; Sigma-Aldrich) in NMM supplemented with 20 ng/ml FGF2 (PeproTech). The cells were kept in FGF2-supplemented medium for 4 days and passaged with dispase upon confluency. After 25 days, throsette structures were dissociated into single neural progenitor cells (NPCs) and passaged using StemPro Accutase (Thermo Fisher Scientific). NPCs can be frozen between days 25–30. On day 35, NPCs were passaged a final time onto plates coated with 1–2 µg/cm^2^ laminin at a density of 5 × 10^4^ cells/cm^2^ in NMM. The cells were then cultured for 2 wk before they were used for experiments or the start of co-cultures with hiPSC-derived microglia.

Astrocytes were generated according to STAR protocol (Perriot et al., 2021) with some modifications, as follows: NPCs age days 25–28 were used for induction of astrocyte differentiation. NPCs were detached using accutase and seeded in mMatrigel-coated 6-wells in astrocyte induction medium (AIM) at a density of 5 × 10^4^ cells/cm^2^. AIM consisted of DMEM/F12 supplemented with 1× N2 supplement, 1× B72 supplement without vitamin A (all from Thermo Fisher Scientific), 10 ng/ml EGF, and 10 ng/ml LIF (both from STEMCELL Technologies). EGF and LIF were added freshly prior to each medium change. AIM was changed every other day for 14 days. Cells were passaged with accutase upon confluency (every 3–4 days) and seeded in Matrigel-coated tissue culture vessels (expanded from 6-wells to T-25 and T-75 flasks) at a density of 5 × 10^4^ cells/cm^2^. Thereafter, cells were switched to astrocyte medium (AM). AM consisted of DMEM/F12, 1× B72 supplement without vitamin A, and 20 ng/ml CNTF (STEMCELL Technologies). AM was changed every other day. Cells were passaged with accutase upon confluency and seeded in Matrigel-coated tissue culture flasks at a density of 4 × 10^4^ cells/cm^2^. After 2 wk in AM, medium was changed twice per week. Cells were passaged with accutase upon confluency and seeded in Matrigel-coated tissue culture flasks at a density of 25 × 10^4^ cells/cm^2^. After 4 wk in AM, the astrocytes were mature and used for experiments. For experiments, astrocytes were seeded at a density of 25 × 10^4^ cells/cm^2^. For further subculturing, CNTF was omitted from the AM, and the medium was changed twice per week. At this point, astrocytes can be frozen.

### Immunocytochemistry (ICC) staining

iPSC and iPSC derived cells were validated by ICC staining as previously described (Fruhwurth et al., 2023). In short, cells grown on Ibidi µ-slides (Ibidi), were washed twice in PBS, fixed in 4% paraformaldehyde for 20 min at room temperature (RT), covered with PBS, and stored at 4°C until analysis. The cells were permeabilized with 0.3% Triton-X100 in TBS for 15 min at RT and incubated with blocking buffer (0.3% Triton-X100 and 5% donkey serum in TBS) for 1 h at RT. Samples were incubated overnight at 4°C with primary antibodies diluted in blocking buffer. Cells were washed three times in TBS and incubated for 1 h at RT with secondary antibodies diluted 1:500 in blocking buffer, washed three times in TBS, counterstained using DAPI, and mounted using Ibidi mounting medium (Ibidi). Samples were imaged using a Nikon A1 inverted confocal microscope.

Primary antibodies used for microglia were IBA1 (17198, dilution 1:500; Cell Signaling), pIRF3 (4947, 1:400; Cell Signaling), and TREM2 (AF1828 1:400; R&D Systems. Has been discontinued); for neurons, Synaptophysin (101 004, 1:250; SYSY), MAP2 (4542, 1:1,000; Cell Signaling), TUJ1 (ab14545, 1:1,000; Abcam), and TBR1 (ab31940, 1:300; Abcam); for astrocytes, GFAP (ab4674, 1:400; Abcam) and S100 (Z0311, 1:400; Dako); and for iPSCs, NANOG (4903, 1:800; Cell Signaling) and OCT4 (2840, 1:400; Cell Signaling).

### Virus stains

For human iPSC-derived CNS cell infections, we used a neurovirulent clinical HSV strain (2762; HSV1) that was isolated from the brain of a patient with fatal HSE during a clinical trial of ACV treatment (Skoldenberg et al., 1984). For all murine experiments, *in vivo* or *in vitro*, we used the HSV-1 McKrea strain (kindly provided by David Leib, Geisel School of Medicine at Dartmouth, Lebanon, NH, USA). HSV-2 (333) was kindly provided by Kristina Eriksson (Department of Rheumatology and Inflammation Research, University of Gothenburg, Gothenburg, Sweden).

HSV-1 and -2 strains were propagated in Vero cells. In short 15 × 10^6^ Vero cells were inoculated with 0.04 MOI for 1 h at 37°C in a humidified CO_2_ incubator. After inoculation, the medium was changed to 18 ml fresh DMEM +2% FCS and 1% Pen/step. Virus was allowed to propagate for 48–72 h, after which the virus was concentrated by centrifugation for 40 min at 4,000 × *g* in Amicon 100-kDa tubes (Merck-Millipore). Viral titer was determined by plaque assay. Influenza A PR/8/34 H1N1 strain was acquired from Charles River.

### RNA isolation, RT-PCR, and qPCR

Human iPSC-derived cells were lysed in RLT Plus buffer (Qiagen) supplemented with 4 mM DTT (Sigma-Aldrich), and RNA was isolated using the RNeasy Mini Kit (Qiagen). cDNA was synthesized using the High-capacity cDNA Kit (Thermo Fisher Scientific). Quantitative PCR was performed using the following TaqMan Gene Expression Assays (Applied Biosystems): *ACTB* (Hs01060665_g1), *18S* (Hs03003631_g1), *IFNB* (Hs01077958_s1), *IL6* (Hs00174131_m1), *TNFA* (Hs00174128_m1), *IL1B* (Hs01555410), *MX1* (Hs00895598_m1), *CXCL10* (Hs00171042), and *ISG15* (Hs01921425). mRNA levels of interest were normalized to the housekeeping gene *ACTB* or *18S* (as indicated) using the ΔΔCt method.

From murine samples (CNS cell cultures and tissue homogenates), RNA was isolated using the High Pure RNA isolation Kit (Roche) according to the manufacturer’s instructions. 90 ng RNA in technical duplicates were used with TaqMan RNA-to-Ct 1-step qPCR mastermix and TaqMan Gene expression assays (Thermo Fisher Scientific); *β-Actin* (Mm00607939), *Ccl2* (Mm00441242), *Cxcl10* (Mm00445235), *Ifnb* (Mm00439552), *Il1b* (Mm00434228), *Il6* (Mm00446190), *Isg15* (Mm01705338), and *Tnfa* (Mm00443260). mRNA levels of interest were normalized to the housekeeping gene *Actb* using the ΔΔCt method.

### Generation of patient-specific mouse strains

Mice carrying the patient-specific R278Q amino acid substitution in IRF3 (C57BL/6J-Irf3<em1Nki>/Nki, MGI:6274685) were generated using microinjection in mouse zygotes isolated from C57BL/6J mice. The injection mixture consisted of Cas9 RNA, a sgRNA targeting exon 6 of the Irf3 gene (5′-GTG​GGA​GTG​GCC​TAG​GCG​CTG​GG-3′) and a ssODN donor template (IDT) to introduce the R278Q point mutation along with two silent mutations L279L and E290E (Fig. S2 A). The mice have the MGI nomenclature; allele: Irf3<em1Nki> (MGI:6274683), strain: C57BL/6J-Irf3<em1Nki>/Nki (MGI:6274685), description: *Irf3* gene—point mutation allele, gRNA(5′-GTGGGAGTGGCCTAGGCGCTGGG-3′) a point mutation in exon 6-R278Q (CGC>CAG) and silent mutations at L279L (CTA>TTG) and at E290E (GAG>GAA).

### Mouse strains

IRF3^−/−^ mice were kindly provided by Ganes C. Sen, Department of Immunology Cleveland Clinic, Cleveland, OH, USA, and IRF3 ^R278Q/R278Q^ mice were bred to use at the animal facility at Aarhus University. WT C57BL/6JRj mice were acquired from Janvier Labs (France) at 5 wk of age and allowed to acclimatize 1 wk prior to start of experiments.

Animals were genotyped by extraction of genomic DNA from tails. At weaning, 3 wk of age, 2 mm of the tail were cut and digested using Invitrogen Platinum Direct PCR Universal Master Mix (Thermo Fisher Scientific). IRF3 R278Q F-primer: 5′-CAT​TTC​TCC​CCT​CTC​ACT​CCA​C-3′; R-Primer: 5′-CTG​TCA​GTT​CCA​TAG​GGT​GCT​C-3′, (LGC Biosearch Technologies), IRF3 KO F-primer d.s.: 5′-GTT​TGA​GTT​ATC​CCT​GCA​CTT​GGG-3′; F-primer: 5′-TCG​TCG​TTT​ACG​CTA​TCG​CCG​CTC​CC-3′; common R-Primer: 5′-GAA​CCT​CGG​AGT​TAT​CCC​GAA​GG-3′ (LGC Biosearch Technologies). Amplification followed a hot start at 95°C for 5 min; 35 cycles were run accordingly: 30 s at 95°C, 15 s at 58°C, and 15 s at 72°C. IRF3^−/−^ genotype was visualized by running the PCR product on a 2% agarose gel at 90 V for 30 min. For the identification of IRF3^WT/R278Q^ and IRF3^R278Q/R278Q^ mice, DNA was extracted from the 2% agarose gel using the Monarch DNA gel extraction kit (New England Biolabs) and eluted in 17 μl. 15 µl of the extracted DNA was added to 2 μl of 10 µM Pr1 primer and commercially sequenced using the LightRun modality from Eurofins Genomics. Sanger sequencing was analyzed using SnapGene software. IRF3^−/−^ and IRF3^R278Q/R278Q^ mice bred at Mendelian ratios, and litters were of average frequency and size compared with Janvier C57BL/6JRj (https://janvier-labs.com/en/fiche_produit/2_c57bl-6jrj_mouse/) (Fig. S2 B). IRF3^−/−^ and IRF3^R278Q/R278Q^ growth were comparable with WT mice and had comparable weight at start of experiments (Fig. S2 C).

### Isolation and culture of brain cell types from newborn mice

Primary microglia, astrocytes, and neurons were isolated from whole brains of P0–3 mice using the Neural Tissue Dissociation Kit (MACS; Miltenyi Biotec) according to the manufacturer’s instructions.

The dissociated cells were plated for 60 min in T175 culture flasks to allow separation of the quickly attaching non-neuronal cell fraction (including microglia and astrocytes) from the neuron containing nonattached cell fraction.

The neuron-containing cell fraction were seeded on poly-L-lysine (Sigma-Aldrich) and laminin (Invitrogen) pre-coated 48-well plates at a density of 2 × 10^5^ cells/well and cultured in Neurobasal-A medium (Gibco) + 2% B-27 Supplement (Gibco), 2 mM L-Glutamine (Gibco), 100 mg/ml Primocin (InvivoGen), and 20 mM floxuridine + 20 mM uridine (Sigma-Aldrich). Medium was changed every third day.

The quickly attaching cell fraction was split into two, one was cultured for microglia enrichment and one for astrocyte enrichment. Both were cultured in DMEM +10% FBS supplemented with 2 mM L-Glutamine and 1% penicillin/streptomycin. Astrocyte enrichment was achieved as previously described (Reinert et al., 2016) by splitting the cultures with 0.5% trypsin +0.5 mM EDTA at a ratio of 1:4 once per week for 4 wk. Microglia enrichment was achieved as previously published (Saura et al., 2003). In short, culturing the confluent mixed glial cells for 4 wk, with medium change once per week without splitting the cells results in microglia growth with layered astrocytes on top. The astrocyte layer growing above the microglia was removed by 30 min mild trypsinization (0.05–0.12% trypsin in DMEM) at 37°C. The strongly adherent microglia remaining are subsequently isolated by trypsinization using 0.5% trypsin +0.5 mM EDTA.

The purity of enriched primary CNS cell cultures was assessed by flow cytometry as previously reported (Dai et al., 2024). Primary CNS cells (astrocytes, microglia, and neurons) were plated 2 × 10^5^ cells/well in 48-well plates and used for experiments.

MBC was obtained as previously described (Katzilieris-Petras et al., 2022). In short, brains were isolated from 0- to 3-day-old mice, and the primary mixed brain culture was achieved by treatment with 2.5% trypsin (Gibco) and subsequently filtered through a 70-µm cell strainer. The cells were seeded in culture medium (DMEM 1640 supplemented with 10% FBS, 100 U/ml penicillin, and 100 μg/ml streptomycin) at a concentration of 8 × 10^5^ cells/24-well.

### Bone marrow–derived macrophages (BMDMs)

For BMDMs, femurs of 8-wk-old male mice were flushed using a 1-ml syringe to isolate the bone marrow. After lysing RBCs by RBC lysis solution (eBioscience), progenitor cells were seeded 5 × 10^6^ cells/dish in 10-cm culture dishes. The cells were cultured for 6 days in 20% L929 medium in 80% DMEM +10% FBS supplemented with 2 mM L-glutamine and 1% penicillin/streptomycin. Medium wash is replaced with fresh 20% L929/80% DMEM every second day. On day 6, BMDM were harvested by trypsinization with 1% trypsin + 0.5 mM EDTA followed by cell scraper release from the culture dish. After wash centrifugation, 5 × 10^5^ cells/well were plated in 24-well plate for experiments.

### Treatments

Human iPSC-derived microglia, astrocytes, and neurons were all plated in 48-well plates at a density of 6 × 10^4^, 2.5 × 10^5^, and 5 × 10^4^, respectively. After seeding the neuron precursors, significant proliferation occurred, and treatment and infection were performed with ∼2 × 10^5^ neurons/cm^2^. Primary CNS cells (astrocytes, microglia, and neurons) were plated 2 × 10^5^ cells/well in 48-well plates and used for experiments. In short, CNS cell cultures were infected with HSV1 (MOI 0.1–10) for 24 h or stimulated with 100 µg/ml 2′-3′cGAMP (InvivoGen) or 25 µg/ml low molecular weight PolyI:C (InvivoGen) for 4 h. For western blot (WB) analysis of IRF3 and phosphorylated IRF3 Ser379, BMDMs were stimulated with 100 µg/ml 2′-3′cGAMP for 2 h. Mixed brain cells were either pre-treated with 25 µM PDTC (HY-18738; MCE) for 2 h or 2 µM BMS-345541 (HY-10518; MCE) for 24 h before HSV-1 infection (MOI 1), 16 h. In separate experiments, microglia cells were depleted from the MBC prior to HSV-1 infection. This was done by treating with 0.5 μM PLX5622 (MCE) for 10 days. PLX5622 is a highly selective brain-penetrant CSF1R inhibitor, allowing extended and specific microglial elimination. The medium was changed every third day. Depletion was confirmed by qPCR of Aif1.

### Immunoblotting

BMDMs were lysed with radioimmunoprecipitation assay (RIPA) buffer (Thermo Fisher Scientific), and the cell lysates were separated by SDS-PAGE, followed by immunoblotting. The blots were developed with SuperSignal West Femto Maximum Sensitivity Substrate (Thermo Fisher Scientific) and ChemiDoc Imaging System (Bio-Rad). The reagents and antibodies used for immunoblotting were XT sample buffer (Bio-Rad), XT reducing agent (Bio-Rad), Precision Plus Protein Dual Color Standards (Bio-Rad), 4–20% Precast Protein Gel (Bio-Rad), NuPAGE MOPS SDS Running Buffer (Thermo Fisher Scientific), PVDF membrane (Bio-Rad), Trans-Blot Turbo Transfer System (Bio-Rad), Skim milk powder (Sigma-Aldrich), anti-Vinculin (V9131; Sigma-Aldrich), anti-IRF-3 (4302; Cell Signaling), anti-pIRF-3 S379 (79945; Cell Signaling), anti-Rabbit IgG-HRP (71-035-152; Jackson ImmunoResearh), and anti-Mouse IgG-HRP (715-036-150; Jackson ImmunoResearch).

### HSV-1 CNS infection in mice

We used an ocular infection route for induction of HSE-like disease to mimic the natural route of HSV-1 entry into the CNS by retrograde transport via the trigeminal ganglion. In short, age-matched (6–8-wk), WT, IRF3^−/−^, IRF3^WT/R278Q^, and IRF3^R278Q/R278Q^ female and male mice were anaesthetized with i.p. injection of ketamine (100 mg kg^−1^ body weight) and xylazine (10 mg kg^−1^ body weight). Using a 29-G syringe, corneas were scarified in a 10 × 10 cross pattern, and mice were inoculated with 2 × 10^6^ PFU HSV-1 McKrea in 5 μl of infection medium (DMEM [Sigma-Aldrich] +2% penicillin/streptomycin [Gibco]) or mock infected with 5 μl of infection medium without virus. Mice were monitored daily for weight loss and development of symptoms. Disease symptoms were scored according to previously published descriptions (Dai et al., 2024). Mice were sacrificed 5 days after infection or if humane endpoints were met, including weight loss >20% of starting weight, skin lesions at eyes, head swelling, severe lethargy, shaking, or unresponsiveness. For kinetics studies, mice were sacrificed on day 0 (uninfected [UI]), 1, 2, 3, 4, and 5 and tissue harvested. For survival studies, mice were followed until reaching humane endpoint or 100% of starting weight (approximately day 10–12). Mice where establishment of HSV-1 infection failed were excluded from analysis. This was defined as no symptoms other than no or only mild eye irritation and no observed weight loss (below 0.00% of starting weight) over the course of the study. Within genotypes, UI and HSV-1–infected mice were littermate controlled.

For treatment experiments, mice (littermates) were randomized to the treatment groups using a random list generator (https://www.randorm.org/lists) after infection and prior to initiation of treatments.

Treatments were initiated on day 3 after infection and administered by i.p. injection 1x daily. All drugs and treatments were diluted to final concentrations corresponding to a volume of 10 μl/g body weight. Drugs were dissolved or diluted in saline (sodium chloride solution, 9 mg/ml [Fresenius Kabi]), which was also used as vehicle control treatment.

ACV (25 mg/ml; Pfizer), treatment dose 50 mg/kg; PDTC, treatment dose 10 mg/kg; BMS-345541, treatment dose 25 mg/kg daily. After dissolving, PDTC and BMS-345541 pulse-sonicated for 5 min prior to administration.

### HSV-2 vaginal infection in mice

Mice were intravaginally inoculated with HSV-2 (333 strain), as previously described in Iversen et al. (2017). In short, 7-wk-old female mice were pre-treated with 2 mg Depo-Provera (Pfizer) by subcutaneous injection 5 days prior to vaginally infection with 6.7 × 10^4^ PFU of HSV-2 333. Mice were monitored, weighted, and scored daily for disease symptoms and survival as described (Iversen et al., 2017). Mice were sacrificed by cervical dislocation when reaching human endpoint (score 4). In separate experiments, vaginal fluids were collected on day 2 p.i. by washing the vagina with 40 μl of PBS, repeated twice. Subsequently the vaginas were isolated, and the tissue processed for RNA extraction and subsequent qPCR using the High Pure RNA isolation Kit (Roche), according to the manufacturer’s instructions, and TaqMan RNA-to-Ct 1-step qPCR mastermix and TaqMan Gene expression assays (Thermo Fisher Scientific).

### Influenza A infection in mice

Mice were anesthetized with 2.5% isoflurane and infected by droplet inhalation via the nose with 1.0 hemagglutinin units of influenza A (Puerto Rico 8/34 H1N1) in 15 μl PBS. Mice were monitored daily for weight loss and development of symptoms of distress. For evaluation of virus titer, mice were sacrificed on day 4 after infection, and the lungs were harvested for quantification of viral RNA by qPCR. For survival studies, mice were followed until reaching humane endpoint (including weight loss >20% of starting weight and signs of distress such as hunched posture, immobility, and strained breathing) or recovery of 100% of starting weight.

### LPS treatment in mice

For *in vivo* assessment of response to LPS, 7-wk-old male and female (littermates) mice were randomly selected to receive i.p. injection with 5 mg/kg LPS from *Escherichia coli* O111:B4 (L2630-10 mg; Sigma-Aldrich) dissolved in physiological saline. Controls received saline. 6-h after treatment, RNA wash was isolated from whole-brain homogenates and analyzed for gene expression by qPCR (procedure described below).

### *In vivo* MRI

5 days after HSV-1 ocular infection, the mice (WT *n* = 6, *Irf3*^*−/−*^*n* = 6, *Irf3*^*R278Q/R278Q*^*n* = 6) were transferred to the preclinical magnetic resonance (MR) scanner facilities, where the terminal MR scan was carried out, similar to previous work (Reinert et al., 2021). Anesthesia was briefly induced with 5% isoflurane in a mixture of medical air (0.4 liters/min) and oxygen (0.4 liters/min) and decreased to 1–2% isoflurane for maintenance during scanning. To facilitate optimal signal-noise-ratio to the brain stem area, the mice were placed in a custom 3D-printed mouse bed (PolyLactic Acid, 3DE premium, 3D Eksperten, Denmark), with modular inserts to accommodate different size mice. Body temperature was measured with a rectal probe (SA Instruments) and maintained at ∼37°C during the scan, with a custom 3D-printed bed insert (Acrylonitrile Styrene Acrylate, 3DE Premium) with integrated tubing for recirculating warm water (Haake SC100 and S5P, Thermo Fisher Scientific). The mice were covered in cloth tissue (Nonwoven swabs, SELEFA; OneMed, Sweden) and fastened to the bed with tape (Micropore, 3 M) to avoid movement-induced image artifacts, and their eyes were covered with ointment (Neutral Ophtha, Ophtha A/S, Denmark). Respiration was monitored (SA Instruments) and maintained (with isoflurane level) at ∼60 breaths per minute.

MRI data were acquired using a Bruker BioSpec 9.4-Tesla MR imaging system, equipped with an 86-mm volume coil (Bruker BioSpin) for transmission and a high-sensitivity rodent cryo-array surface coil (Bruker BioSpin) for reception. Localizer scans were performed to ensure optimal animal positioning, followed by a B0-field map acquisition (echo time [TE]: 1.84 ms, echo spacing: 3.57 ms, repetition time [TR]: 20.0 ms, field-of-view [FOV]: 32 × 32 mm, and matrix size: 128 × 128) for shimming. Scan time was 5 min 28 s.

Next, a high-resolution structural scan was acquired, using a T2 TurboRARE sequence (FOV: 10 × 10 mm; matrix: 200 × 200), resulting in an in-plane resolution of 50 × 50 µm and a coronal slice thickness of 250 µm (18 slices in total). The slice package was angled to accommodate the curvature of the brain stem region. Scan parameters were effective TE: 20 ms, TR: 2,300 ms, 12 averages, and RARE factor: 4. Scan time was 23 min. In a few animals, an additional lower resolution structural scan was obtained to aid the manual segmentation. Modified scan parameters were TE: 33 ms, matrix: 100 × 100, with 9 coronal slices at a thickness of 500 µm. Scan time was 11 min 30 s.

### Preprocessing of MR data and volume of interest delineation

The structural images were denoized using a custom adapted algorithm (MATLAB; The MathWorks) using windows of 2 × 2 voxels (Veraart et al., 2016). Blinded to genotype and infection status, high-resolution raw and denoized images were visually inspected by segmentation reader (MHV). Utilizing Horos software (Horosproject.org), pathological lesions in the brain stem were delineated, and lesion volumes were calculated as number of voxels in the delineated volumes multiplied by the nominal voxel size.

### Isolation and analysis of biological material from mice

Eye wash was collected on day 2 after infection. Eye wash was performed under brief isoflurane anesthesia by gently wiping a sterile cotton swab in circular motion thrice around and twice across the cornea of a slightly proptosed eye. Repeating the motion with a new cotton swab on the second eye. The swabs were stored in 0.5 ml DMEM +1% penicillin/streptomycin and kept at −80°C until viral titer was determined. Eye wash was only performed in experiments of viral spread specifically.

For isolation of eyes, TG, and brain stem, mice were anesthetized with 3% isoflurane and culled by decapitation. Eyes, brain stem, and TG were dissected out, snap-frozen, and kept at −80°C until use.

Tissues were added 200–500 μl DPBS (Sigma-Aldrich) and added one 3-mm Tungsten Carbide Bead (Qiagen)/sample and homogenized for 3 min, frequency of 30/s in a TissueLyzer II (Qiagen). 50 μl tissue homogenate were used for RNA extraction using a High Pure RNA isolation kit (Roche). RNA concentration was measured using a NanoDrop.

For mesoscale and WB, 150 μl tissue homogenate were used lysed in RIPA buffer (Bio-Rad) + PhosSTOP (Roche) + mComplete (Roche) and snap-frozen on dry ice. The remaining homogenate were centrifuged for 10 min at 3,000 × *g* at 4°C, pelleting of cellular debris. The cleared supernatant containing virus was used for virus plaque assay to determine the viral titer.

### Quantification of virus

Live virus titers were determined by plaque assay or tissue culture infective dose 50% assay (TCID50%). In short, eye, TG, and brain stem homogenates were centrifuged for 10 min at 3,000 × *g* precooled to 4°C to pellet cellular debris.

For plaque assay, the cleared homogenate or cell culture supernatant was titrated in duplicate onto a monolayer of adherent Vero cells (ATCC) in 6-well plates and incubated for 1 h at 37°C, tilting the plates every 15 min for equal distribution and viral adsorption. 0.4% IgG was added after incubation to neutralize any viral progeny released into the medium during infection, allowing virus to spread via cell-to-cell contact, resulting in plaque formation in the cell layer. 72 h after incubation, the cell layer was fixed and stained with methylene-ethanol stain, and plaques were counted.

For quantification of viral titer in vaginal washes and iPSC-derived cell cultures, we used the TCID50% assay. In short, washes were serial diluted 10-fold (10^−1^–10^−11^) and added in octuplicates to a monolayer of adherent Vero cells in 96-well plates and incubated 48–72 h at 37°C. Cytopathic effect was evaluated by microscopy using a light microscope (Leica DMi1), and the viral titer (TCID50%/ml) was calculated using the Reed–Muench method (Dai et al., 2024; Reinert et al., 2016).

Viral transcripts were quantified by qPCR using the following primers and probes:

HSV-1 gB (forward: 5′-GCA​GTT​TAC​GTA​CAA​CCA​CAT​ACA​GC-3′; reverse: 5′-AGC​TTG​CGG​GCC​TCG​TT-3′; probe: 5′FAM-CGGCCCAACATATCGTTGACATGGC-BHQ-1′3′); HSV-2 gB (forward: 5′-TGC​AGT​TTA​CGT​ATA​ACC​ACA​TAC​AGC-3′; reverse: 5′-AGC​TTG​CGG​GCC​TCG​TT-3′; probe: 5′FAM- CGCCCCAGCATGTCGTTCACGT-BHQ1′3′).

IAV M protein (F: 5′-AGA​TGA​GTC​TTC​TAA​CCG​AGG​TCG-3′; R: 5′-GCAAAAACATCTTC AAGTCTCTG-3′; probe: 5′FAM-TCAGGCCCCCTCAAAGCCGA-BHQ-1′3). All viral primers and probes were obtained from LGC Biosearch Technologies.

### Suspension mass cytometry (CyTOF)

Animals were anesthetized with ketamine and xylazine and perfused through the left heart chamber (and draining from the right) with a minimum of 25 ml DPBS. Brain stems were surgically removed and transferred to individually labeled 2-ml Eppendorf tube containing 1.5 ml digestion buffer (1 mg/ml Collagenase I [Gibco] + 10 U/ml Pulmozyme [Dornase alfa, Roche] + 1% penicillin/streptomycin [Gibco] in RPMI). The brain stems were finely cut into 1–2-mm^3^ pieces using small surgical scissors. Tissue fragments were placed on a continuous rotation device in an incubator at 37°C with 5% CO_2_ and digested for 40 min.

Following incubation, 20 μl 0.5 M EDTA (stock solution), final concentration 10 mM, was added to the 2-ml tubes to stop the enzymatic reaction and resuspended by pipetting up and down with a 1-ml pipette. Single-cell suspensions were achieved by passing the resuspended digest through a nylon mesh (70-μm pore size; Corning) into a 50-ml tube and washing with 25 ml 2% FBS-HBSS. Single-cell suspensions were centrifuged for 8 min (350 × *g*) at 4°C. Supernatant was removed by aspiration, and the cell pellet was resuspended in 10 ml 37% Percoll (Sigma-Aldrich) density gradient medium in PBS. Gently layered 5 ml of PBS on top of the cell/gradient layer, creating a phase separation. The gradient was centrifuged for 20 min (2,500 × *g*) at RT (acc 1, brake 0) to layer the myelin debris on top and pellet the cells. The cell pellet was washed with 2% HBSS-FBS and centrifuged for 8 min (350 × *g*) at 4°C and finally resuspended in 500 μl D-PBS/0.2% BSA, filtered through a 40-μm mesh filter (Corning), and counted.

Up to 3 × 10^6^ cells/sample were washed with PBS, incubated with Cisplatin for dead cell exclusion (Cell-ID Cisplatin; Standard BioTools), (0.25 μM final concentration, 5-min incubation at RT), quenched with Cell Staining Buffer (CSB; Standard BioTools), and subjected to Palladium-based barcoding following the manufacturer’s protocol (Cell ID 20-Plex Pd–Barcoding Kit; Standard BioTools). Barcoded cells were washed, pooled, counted, and resuspended in CSB at a concentration of 3 × 10^6^ cells/100 μl. Before immune staining, cells were incubated for 10 min with purified rat anti-mouse CD16/CD32 (Mouse Fc Block; BD). Cell surface staining was conducted following the manufacturer’s protocol (Maxpar Cell Surface Staining with Fresh Fix protocol; Standard BioTools). See Table S1 for full antibody panel information.

Metal-labeled Abs were obtained from Standard BioTools, except Siglec-F (BD), which was custom labeled using the MaxPar X8 labeling kit (Standard BioTools) according to the manufacturer’s instructions (Standard BioTools). After staining and washing, the cells were fixed with 1.6% formaldehyde for 15–30 min and DNA-stained overnight at 4°C with 250 nM of Cell-ID DNA Intercalator-IR (Standard BioTools) in FixPerm Buffer (Standard BioTools). On the next day, cells were washed once with CSB, twice with Cell Acquisition Buffer (Standard BioTools), counted, and kept as pellet at 4°C. Immediately before sample acquisition, cells were resuspended in EQ Four Element Calibration Beads (Standard BioTools) diluted in Cell Acquisition Buffer at a concentration of 1 × 10^6^ cells/ml. Sample acquisition was performed on a CyTOF Helios instrument (Standard BioTools) at the Aarhus university mass cytometry unit.

CyTOF datasets were exported as FCS files, randomized, normalized, concatemerized, and de-barcoded according to the manufacturer’s instructions (CyTOF software v7; Standard BioTools). FCS Express (v7; DeNovo Software) was used for preparation of the data for the subsequent data analysis pipeline. In short, Single-data files were cleaned using Gaussian distribution parameters and removal of EQ-beads (Standard BioTools), subsequently gated on DNA-positive (Ir positive), viable (Cisplatin neg), Ter119-negative events, and finally a CD45-positive or CD11b-positive Boolean gate was used to export the cleaned data files.

Subsequently the following R-packages were used for further data processing: cytutils (v0.10) for renaming of the metal channel annotations: flowCore (v2.14.2) for reading in the datafiles (Hahne et al., 2009); catalyst (v1.26.1) for constructing the SingleCellExperiment (sce); flowSOM (v2.10.0) for cell population clustering (Van Gassen et al., 2015); ConsensusClusterPlus (v1.66.0) for cluster merging and annotation (Wilkerson and Hayes, 2010); catalyst (v1.26.1) for Uniform Manifold Approximation and Projection (UMAP v0.2.10.0) analysis (McInnes et al., 2020, *Preprint*); the resulting sce was used for subsequent proportion calculations graphically visualized using GraphPad Prism.

### RNAseq

Brain stems were harvested on day 5 after infection. Animals were sedated with ketamine and xylazine and perfused with a minimum of 25 ml of PBS. 2–3 brain stems were pooled as one sample. Brain stems were cut into pieces and digested for 40 min at 37°C with 5% CO_2_ in PBS supplemented with 1 mg/ml Collagenase 1 (Roche), 10 U/ml Pulmozyme (Dornase alpha), and 3.423 mg/ml Trehalose (Sigma-Aldrich). 20 μl of sterile EDTA was added to each sample and incubated at 37°C for another 5 min. Tissues were then mechanically disrupted through a 70-μm cell strainer into a single-cell suspension. Single-cell suspensions were washed with 25 ml 2% FCS-HBSS (Thermo Fisher Scientific), centrifuged, and resuspended in a mixture of PBS with 0.5% BSA (VWR) and Debris Removal Solution (Miltenyi Biotec). After overlaying PBS with 0.5% BSA, suspensions were centrifuged and resuspended, and the cell count was adjusted to 10^6^ cells/ml. Single-cell suspensions were converted to barcoded scRNAseq libraries following the manufacturer’s instructions of Chromium Single Cell 3′ Library, Gel bead & index kit, and Chip G Kit (v3.1; 10x Genomics), aiming for recovering 10,000 cells per library. Quality control of libraries was performed with TapeStation 4200. After QC, libraries were sequenced on an MGI DNBSEQ-G400 sequencer.

### Analysis of scRNAseq data

Reads obtained from the DNBSEQ-G400 were aligned to a composite reference genome containing the mouse genome and HSV-1 genome using Cell Ranger v7.1 by 10x Genomics. The unique molecular identifier count matrices generated were then converted into Seurat objects using the Seurat package (v4.4) for subsequent analyses. Cells with between 200 and 5,000 detected genes and <30% mitochondrial gene content were selected for further analysis. Normalization was conducted using sctransform (v2). To correct batch effects and ensure data uniformity across samples, we applied CCA integration. This was followed by dimensionality reduction through PCA and UMAP. The KNN method was then used to cluster the integrated count matrices, simplifying the dataset for visualization and enabling the identification of distinct cellular populations. WT UI samples were collected (from ref (Ding et al., 2025) for compositional comparison of monocyte subpopulations.

The identification of differentially expressed genes (DEGs) among various conditions, cell types, and subpopulations was achieved using the FindMarkers function, after the correction of counts by the PrepSCTFindMarkers function prior to DEG analysis. Identified upregulated DEGs with an adjusted P value of 0.01 or lower were subjected to over-representation analysis against gene ontology (GO). The R package clusterProfiler (v4.6) was employed.

Using NicheNet (v2.1), we identified prominent ligand–receptor interactions that were highly expressed and active between monocyte #0 and various other cells. To explore the downstream impacts further, we analyzed the enrichment of predicted target genes within the receiver cell types, thus inferring the signaling target genes of the communication pairs.

Publicly available datasets of single-cell transcriptomes from Japanese encephalitis virus-infected mouse brains (GEO: GSE237915) were integrated with our dataset via Seurat’s anchor-based data transfer workflow (v4.3). First, transfer anchors between the reference and our cells were identified with FindTransferAnchors. Subpopulations annotations from the reference were then propagated to the query cells using MapQuery, and each cell was assigned the label with the highest prediction score. These transferred labels defined the subpopulations and were used in summary statistics.

### Assessment of BBB integrity

A 2% solution of Evans Blue in normal saline (4 ml/kg of body weight) was injected i.p. The stain was allowed to circulate for 4 h. Afterward, the mice were transcardially perfused with 25 ml of PBS and fixed in 4% formaldehyde. Brain stems were dissected into 5-mm coronal sections. Evans blue dye leakage into the mouse brain stems from ventral position (uncut) and coronal sections were imaged on Leica M165FC microscope, using a 2× and 3.2× objective, respectively.

### Immunohistochemistry

Animals were anesthetized with ketamine and xylazine and perfused through the left heart chamber (and draining from the right) with a minimum of 25 ml DPBS followed by 20 ml 10% formalin solution (Sigma-Aldrich). The fixed brains were dissected out and transferred to individually labeled tubes containing 10% formalin for continued fixation for 24 h. Formalin fixed brains were paraffin embedded (FFPE) in histology blocks. FFPE tissue blocks were cut into slides of 6 µm using a SLEE Microtome (SLEE Medical) and transferred to Superfrost Plus Microscope slides (Thermo Fisher Scientific).

For immunofluorescence and confocal microscopy, FFPE slides were rehydrated by heating for 30 min at 60°C following rapid transfer to a xylene bath for 2 × 5 min. The rehydration followed incubation for 2 × 5 min at decreasing concentrations of ethanol, starting at 99% >96% >70%. After the last incubation with ethanol, the slides were washed under running demineralized water for 5 min. The rehydrated slides were soaked in prewarmed (80°C) Dako Antibody Target Retrieval Buffer (Dako) and incubated in oven at 80°C for 30 min, followed by two time washing in cold DPBS and cold blocking of endogenous peroxidase using methanol for 30 min on ice. Slides were washed two times for 5 min in PBS, followed by rinsing for 10 min in Tris-buffered Saline +0.3% Triton X 100 +1% BSA (Sigma-Aldrich). Slides were incubated with primary antibodies (mouse Anti-HSV1 + HSV2 ICP5 Major Capsid Protein (Clone 3B6; Abcam), rat anti-mouse CD45 (clone 30-F11; eBioscience), Rabbit anti-Cleaved Caspase 3 asp175 (clone 5A1E; Cell Signaling Technology), or polyclonal goat anti-mouse Iba1 (ab5076; Abcam) at 1:500 dilution in DPBS+1% BSA at 4°C overnight in a humidified chamber. The next day, slides were temporized for 1 h at RT, followed by rinsing 3× 3 ml/slide with TBS-T + 0.2% BSA. Slides were incubated for 1 h at RT with Alexa-Flour 568–conjugated secondary donkey anti-mouse and Alexa-Flour 488 Donkey anti-rabbit IgG, respectively (Thermo Fisher Scientific), and were diluted 1:500 in TBS-T + 1% BSA. Slides were at last washed 3 × 3 ml/slide with TBS-T + 0.2% BSA followed by soaking 10 min in PBS. Cover glasses were mounted using 10 μl Prolong Gold (Thermo Fisher Scientific).

Slides were imaged using a Zeiss LSM 800 Airyscan Laser scanning confocal microscope. Both the right and left side of the brain stem (UI *n* = 4, WT *n* = 6 and IRF3^R278Q/R278Q^*n* = 5) at 2 different anatomical locations (−5.80 and −6.34 mm, relative to bregma, respectively) were imaged at 20× and 40×. Images at 20× were used for automated quantification using FIJI (ImageJ) software. In short, channels were split, and auto contrast was set, followed by application of a Tophat filter, radius 10, and auto threshold (triangle). A mask was created, and cells were discerned by applying the watershed binary. Individual automated ROIs were counted for each image. Only images containing HSV1 foci were quantified. In short, all nucleated (DAPI^+^) cells, all CD45^+^ cells, all HSV1^+^ cells, and all apoptotic cells (Cleaved caspase-3^+^) were counted/image.

### Statistics and reproducibility

The data are represented as means of biological replicates ± SD. The sample number (*n*) indicates the number of cellular experimental repeats, biological replicates, mice, or images included in quantification as specified in the figure legends.

Statistical significance between two groups was assessed using two-tailed unpaired *t* tests for normally distributed data. For multiple comparisons involving one or more variables, two-tailed one-way or two-way ANOVA tests were employed, respectively. When ANOVA results indicated significant differences between group means, post hoc two-tailed unpaired *t* tests were conducted to compare specific groups of interest. Survival analysis was performed using the log-rank Mantel–Cox test, and disease progression and viral replication kinetics over time was analyzed using mixed-effect analysis with Geisser-Greenhouse correction for multiple interacting variables (time and genotype). The reported P values thus reflect whether disease progression—or viral load in various tissues—differs significantly between genotypes over time. Disease progression data are presented with error bars for SEM. Data presented represent single experiments with three or more biological replicates unless specified in the figure legends. Each experiment was repeated at least three times with consistent results, unless noted otherwise. P values <0.05 were considered statistically significant, *P < 0.05, **P < 0.01, and ***P < 0.001.

### Online supplemental material

Fig. S1 shows the immunofluorescence image validation of iPSC cultures and iPSC-derived cortical neurons, microglia, and astrocytes, and their respective type I IFN response to HSV-1 infection and PRR activation. Fig. S2 shows the characterization of the transgenic mouse carrying the IRF3 R278Q gene variant, the cellular CNS response to HSV-1 infection and PRR activation, and disease susceptibility of mouse models of pulmonary IAV or vaginal HSV-2 infection. Fig. S3 contains a comparative analysis of murine systemic and CNS responses to HSV-1 infection *in vivo* (serum and brain stem) and *in vitro* MBCs and BMDMs. Fig. S4 presents extended data analysis of recruitment of myeloid cells into the HSV-infected *Irf3*^*R278Q/R278Q*^ brain, from CyTOF/mass cytometry and scRNAseq. Fig. S5 shows the extended characterization of inflammatory monocyte population enriched in *Irf3*^*R278Q/R278Q*^ mice, including NicheNET analysis of communication between the identified inflammatory monocyte population and cells of the CNS. Table S1 is a list of metal-conjugated antibodies used for mass cytometry analysis of cells from HSV-1–infected mouse brain stems.

## Supplementary Material

Table S1 shows the CyTOF surface marker antibody panel.

SourceData FS2 is the source file for Fig. S2.

## Data Availability

All data needed to evaluate the conclusions in the paper are present in the paper or the supplemental materials. The scRNAseq data from HSV-1–infected brain stems have been made publicly available under GEO accession no. GSE305693, and scRNAseq data from UI WT control brain stems are available under GEO accession no. GSE283518. Single-cell transcriptomes from JEV-infected mouse brains are available under GEO accession no. GSE237915. Further information and requests for resources and reagents should be directed to and will be fulfilled by the corresponding author.
