# Supplementary material for: Role for NF-κB in herpes encephalitis pathology in mice genocopying an inborn error of IRF3-IFN immunity
Source: J Exp Med. 2025 Oct 9;223(1):e20250064. doi: 10.1084/jem.20250064 (PMC12510166; doi:10.1084/jem.20250064)
Supplement: Table S1 — shows the CyTOF surface marker antibody panel. [file jem_20250064_tables1.docx]

Table S1: CyTOF surface marker antibody panel

| **Antibody** | **Metal label** | **Clone** | **Company** |
| --- | --- | --- | --- |
| BB220 | 176Yb | RA36B2 | Standard Biotools |
| CD115 | 144Nd | AFS98 | Standard Biotools |
| CD11b | 148Nd | M1_70 | Standard Biotools |
| CD11c | 142Nd | N418 | Standard Biotools |
| CD14 | 156Gd | Sa 14-2 | Standard Biotools |
| CD19 | 149Sm | 6D5 | Standard Biotools |
| CD206 | 169Tm | C068C2 | Standard Biotools |
| CD24 | 150Nd | M1_69 | Standard Biotools |
| CD326 | 166Er | G8.8 | Standard Biotools |
| CD36 | 147Sm | HM36 | Standard Biotools |
| CD38 | 175Yb | 90 | Standard Biotools |
| CD3e | 152Sm | 1452C11 | Standard Biotools |
| CD4 | 145Nd | RM4-5 | Standard Biotools |
| CD43 | 146Nd | S11 | Standard Biotools |
| CD45 | 089Y | 30F11 | Standard Biotools |
| CD62L | 160Gd | MEL14 | Standard Biotools |
| CD64 | 151Eu | X54-5_7.1 | Standard Biotools |
| CD86 | 172Yb | GL1 | Standard Biotools |
| CD8a | 168Er | 53_6.7 | Standard Biotools |
| CX3CR1 | 164Dy | SA011F11 | Standard Biotools |
| F480 | 159Tb | BM8 | Standard Biotools |
| Ly6C | 162Dy | HK1.4 | Standard Biotools |
| Ly6G | 141Pr | 1A8 | Standard Biotools |
| MHCII | 174Yb | M5_114.15.2 | Standard Biotools |
| NK1_1 | 165Ho | PK136 | Standard Biotools |
| SiglecF | 153Er | E50-2440 | BD |
| TCRb | 143Nd | H57-597 | Standard Biotools |
| TER119 | 154Sm | TER119 | Standard Biotools |
