# Supplementary figures and images for: Role for NF-κB in herpes encephalitis pathology in mice genocopying an inborn error of IRF3-IFN immunity
Source: J Exp Med. 2025 Oct 9;223(1):e20250064. doi: 10.1084/jem.20250064 (PMC12510166; doi:10.1084/jem.20250064)

Fig. S2F

MW

45 kDA

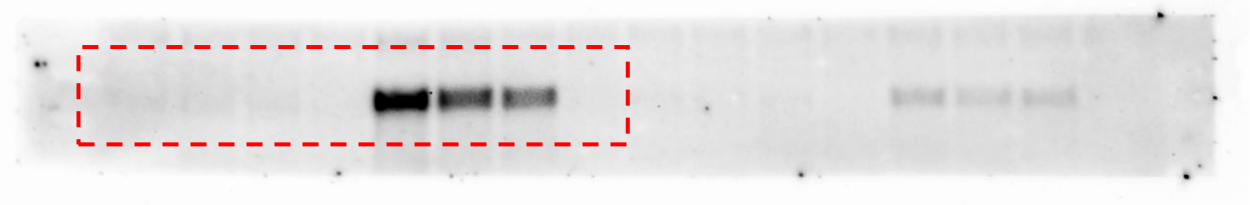

IRF3 pS379

50-55 kDA

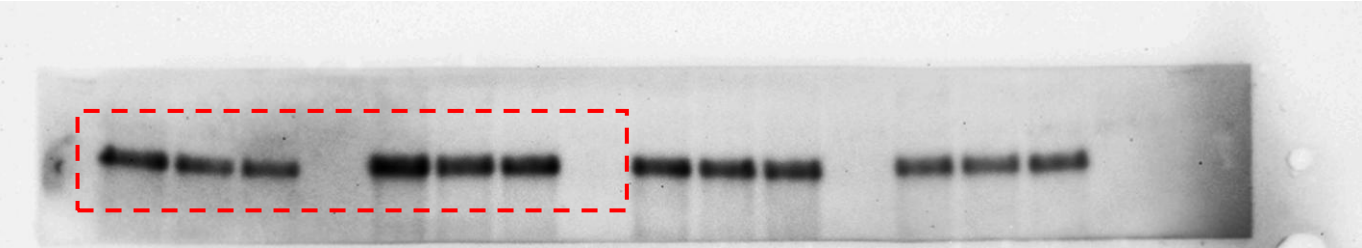

IRF3

124 kDA

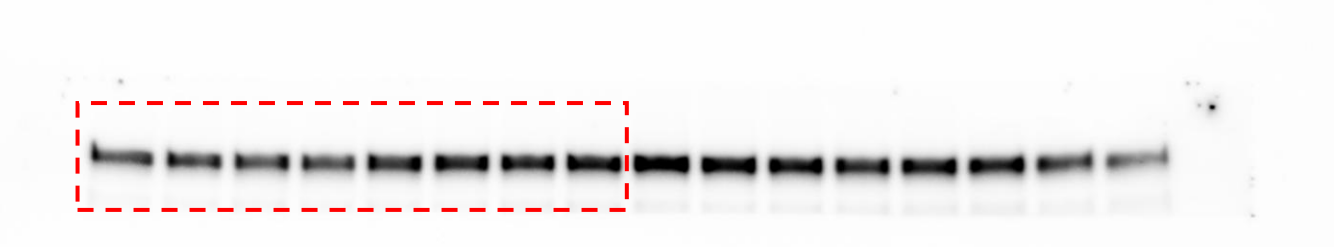

Vinculin

Supplement: SourceData FS2 — is the source file for Fig. S2. [file jem_20250064_sourcedatafs2.pdf]
